# Incentives, Health, and Retirement: Evidence From a Finnish Pension Reform

**DOI:** 10.1002/hec.4917

**Published:** 2024-12-23

**Authors:** Joonas Ollonqvist, Kaisa Kotakorpi, Mikko Laaksonen, Pekka Martikainen, Jukka Pirttilä, Lasse Tarkiainen

**Affiliations:** ^1^ Finnish Institute for Health and Welfare Helsinki Finland; ^2^ Finnish Centre of Excellence in Tax Systems Research (FIT) Tampere Finland; ^3^ Tampere University Tampere Finland; ^4^ Finnish Centre for Pensions Helsinki Finland; ^5^ University of Helsinki Helsinki Finland; ^6^ Max Planck Institute for Demographic Research Rostock Germany; ^7^ Max Planck – University of Helsinki Center for Social Inequalities in Population Health Helsinki Finland; ^8^ VATT Institute for Economic Research Helsinki Finland

**Keywords:** health, incentives, pension reform, retirement

## Abstract

This paper examines, using exogenous variation generated by a Finnish pension reform implemented in 2005, the interplay between health and financial incentives to postpone retirement. Based on detailed administrative data on individual health and retirement behavior, we focus on whether individual reactions to incentives vary according to health status and analyze whether individuals with ill health are also able to take advantage of the potential monetary benefits of delayed retirement created by the reform. We find that on average, individuals react to the financial incentives created by the reform as expected. This result holds for most of the health‐related subgroups we analyze. However, those with a long period of sickness absence are less likely to respond to changes in the financial incentives to postpone retirement.

## 1 Introduction

Many countries faced with the challenge of population aging have implemented pension reforms with the objective of extending working lives and improving the sustainability of public finances (OECD 2019). A key tool in these reforms has been to provide financial incentives for individuals to postpone retirement.

In addition to the question of whether such incentives work on average, an important and yet underexplored issue relates to potential heterogeneity in individual responses, and the associated equity implications of these types of reforms. This paper focuses on one particular dimension of heterogeneity, which is highly relevant for the elderly population, namely health.

Earlier empirical evidence indicates that ill health is a key factor behind early old‐age retirement (e.g., van Rijn et al. 2014 and Leijten et al. 2015). We examine whether incentives inherent in pension reforms exacerbate this pattern: We ask whether such incentives, and the associated monetary benefits, can only be utilized by persons who have relatively good health and are therefore better positioned to continue working. Further, because of a positive correlation between health and socioeconomic status, the benefits of working longer may then be reaped primarily by high‐income individuals. A new kind of efficiency‐equity trade‐off may therefore arise: improving incentives could increase the employment of older workers, but it may also increase inequality among them if employment improvements concentrate on healthy high socioeconomic status individuals.

While the impact of retirement incentives on the decision to continue working has been examined by a large number of earlier studies—we discuss the literature below—ours is one of the first papers on this potential trade‐off. We conduct our analysis in the context of a Finnish pension reform implemented in 2005. This was a major nationwide reform that influenced retirement incentives very differently across the population, depending for example on the individual's age and accrued pension. The reform therefore enables us to utilize exogenous variation in the incentives to retire. In addition to data on retirement behavior, we utilize detailed administrative data on various individual health measures. A key focus in this paper is the potential heterogeneity in individual reactions to retirement incentives. If individuals react differently to these incentives, and if the strength of the reaction is correlated with one's health, the reform could indeed lead to adverse equity consequences.

Overall, the way individuals react to incentives is a huge question in economics. Individuals may differ in how they make economic decisions for many reasons, for example due to differences in time preferences (Tanaka, Camerer, and Nguyen 2010), risk preferences (Gloede, Menkhoff, and Waibel 2015), liquidity constraints (Carvalho, Meier, and Wang 2016) or self‐control (Bernheim, Ray, and Yeltekin 2015). Potential differences in decision‐making in terms of health (Decker and Schmitz 2016) and cognitive abilities (Dohmen et al. 2010; Mani et al. 2013) have also been analyzed.

In our context, a connection between health and reactions to incentives may arise for various reasons. First, individuals with ill health may simply be unable to postpone retirement, and their reactions to incentives may be muted for this reason. However, many of the health conditions captured by our data are such that they do not necessarily prohibit working per se. Second, reacting to tax‐benefit policies in an optimal way often requires difficult financial calculations and long‐term planning. If an individual's attention is drawn to other problems associated with one's current life situation, the ability to engage in long‐term planning may be hindered. Shah, Mullainathan, and Shafir (2012) discuss this issue in the context of poverty; we conjecture that a similar mechanism may be operational if an individual is preoccupied with health problems. Finally, a factor that points in the other direction is the following: to compensate for the greater opportunity cost of working, individuals with ill health may in fact require larger financial returns for continuing to work and may therefore respond more strongly to improved incentives. The pattern of heterogeneity that we should expect to see is therefore not clear a priori.

Our results indicate that on average individuals react to retirement incentives in the expected manner: The better the financial incentives to postpone retirement are, the more likely the individual is to postpone retirement. Furthermore, many types of individuals appear to react to retirement incentives, and it therefore does not seem to be the case that the ability to take advantage of better incentives is limited to any specific group. On average, less healthy individuals retire earlier, as expected, but our results do not indicate strong and consistent differences in reactions to incentives between population groups defined using health indicators related to having received treatment in specialized care or purchases of prescribed medication. However, we do find that individuals with a spell of sickness absence in the previous year react to incentives less strongly than other groups. These results are important, given that sickness absence is a health measure that is directly related to the ability to work. The results suggest that individuals who have work related health conditions for which they have not received effective treatment—in the sense that the illness has lead to sickness absenteeism—are less likely to respond to financial incentives to postpone retirement than their more healthy peers.

In the main analysis, we focus on transitions to old‐age pension, but the results are robust to including disability pension recipients, an important group when considering the linkages between health and retirement, into the analysis. Also a whole host of other robustness checks are carried out and the main results stay similar across specifications.

The analysis in this paper connects to the larger literature on the effects of pension reforms on retirement behavior. The effects of incentives on retirement have been studied for example in Furgeson, Strauss, and Vogt (2006), Coile and Gruber (2007), Hanel (2010), Brown (2013), Johansson, Laun, and Palme (2014), Manoli and Weber (2016), Hernæs et al. (2016) and Engels, Geyer, and Haan (2017). Some of them find strong responses to retirement incentives, but the results vary considerably. van Rijn et al. (2014) and Leijten et al. (2015) studied the connection between health and retirement behavior. They found that ill health increases the likelihood of early old‐age retirement, but these papers did not analyze reactions to retirement incentives per se.^1^ Kerkhofs, Lindeboom, and Theeuwes (1999) estimate the impact of incentives on the retirement decision, while at the same time controlling for health, without examining the interaction between health and incentives. Staubli and Zweimüller (2013) examine, in turn, the employment impacts of increases in the early retirement age utilizing a policy reform in Austria. They find that the employment increases were greater among those with better health (measured by sickness absence), whereas workers with worse health found other pathways to retirement, such as disability benefits. Their paper examines responses to age‐based eligibility rules, rather than monetary incentives, which is in our focus. A theory model of health and retirement is built by Garcia‐Gomez et al. (2017). Their model predicts that wealthier individuals (compared to poorer individuals) are more likely to retire for health reasons and that health problems make older workers more responsive to financial incentives. When examining empirically the interaction effect of health and retirement incentives they focus on health shocks, defined as unpredicted hospitalisations.

Our paper contributes to the literature in several ways. We exploit exogenous changes created by a pension reform, and provide one of the first studies offering a systematic analysis of potentially heterogeneous reactions to the reform by people with different health status. We use high‐quality administrative data on health, covering a wide variety of health variables, such as mental health and sickness absence. All our health indicators are objective measures (as opposed to self‐reported health status) and lagged by 1 year, mitigating the potential endogeneity of health on labor market outcomes. The benefit of working with a larger set of health measures is that by doing so we can cover key dimensions of health that are also significant determinants of retirement.

Earlier analysis of the Finnish reform of 2005 has been provided by Uusitalo and Nivalainen (2013), who examined the mean response to the reform and found relatively strong effects of the incentives created by the reform on retirement decisions. They did not analyze the potential heterogeneity in the reactions to the reform, which is crucial for understanding the associated efficiency‐equity trade‐offs. Leinonen et al. (2016) on the other hand provide descriptive evidence showing that healthier individuals start to retire earlier after the reform. They, however, do not attempt to provide causal estimates of the effects of the reform or analyze how incentives affect retirement decisions at the individual level. Gruber, Kanninen, and Ravaska (2022) also analyzed the main effects of the reform, with a focus on the effect of the change in the statutory retirement age, which was another key feature of the reform. We control for the effect of age limits in our analysis. The unique contribution of our study is that we focus on differential reactions to incentives. In particular, we provide a comprehensive assessment of the effects of changes in retirement incentives on retirement decisions brought about by a major national pension reform. We focus on older workers, a population where health differences are prominent, to assess heterogeneous effects of the reform on different types of individuals.^2^

The rest of the paper is organized as follows. Section 2 introduces the Finnish pension system and the 2005 reform. Section 3 presents the empirical strategy. Preliminary results are presented in Section 4, and the robustness of the findings is discussed in Section 5. Conclusions are offered in the final section.

## 2 The Finnish Pension System and the 2005 Reform

The Finnish pension system has two elements: (1) earnings‐related pensions and (2) residence‐based national pensions. Participation in the earnings‐related pension system is mandatory and covers virtually all earnings and workers. The level of an individual's pension is determined by her working history, the earnings received and age at retirement. National pensions and the so‐called guarantee pension (introduced in 2011 to guarantee a minimum level of income to all pensioners living in Finland) are proportional to the earnings‐related pension. They are paid to those individuals who have a low accrued pension. Each euro of accrued earnings‐related pension cuts national pensions by 50 cents and the maximum amount of the national pension was 529.68 euro per month in 2005. Also, the marital status of the individual affects the amount of the national pension.

The Finnish pension system has statutory retirement ages for full and early old‐age pensions, explained in more detail below. In addition to these two, there are several alternative retirement paths, which differ in their eligibility criteria. The different pathways include part‐time pension, disability pension, and unemployment pension (abolished in the 2005 reform).

The reform implemented in 2005 implied major changes to the key parameters of the pension system (age limits, accrual rates, etc.). The first reform laws were passed in the middle of 2003, and the new rules took effect as of January 2005. An information campaign about the reform was implemented already at the beginning of 2004.

The key feature of the reform for our analysis is that the new rules implied different changes in the incentives to retire for different groups of individuals. In short, the reform created variation in retirement incentives between population groups and over time, and this variation can be utilized to estimate the causal effects of the reform. Below, we describe the changes implemented in the reform in detail.

Table 1 shows the main features of the 2005 pension reform. Before 2005, the full (or default) retirement age (FRA) was 65 years. Early old‐age retirement (ERA) was possible from age 60 onwards. Accrued pensions were cut by 0.4% for each month of early retirement before the age of 65. Delaying retirement after the age of 65 increased the accrued pension by 0.6% for each month. The level of pensions was calculated based on earnings for the last 10 years before retirement for most individuals, although the rules were different for job switchers. The measure of accrued pension that we use in the analysis is calculated by the Finnish Centre for Pensions, and accounts for these complications. It was limited to 60% (66% for public‐sector workers) of the highest annual salary for those years. Some public sector workers also had different retirement ages depending on their occupation. Pensions started to accrue at the age of 23 and the accrual rate until age 59 was 1.5%. For 60‐ to 65‐year‐olds, the accrual rate was 2.5%. A so‐called halfway index^3^ was used to convert accrued pensions to the retirement year's money and to adjust pensions paid to those under 65 years old. For those over 65 years old, the earnings‐related pension index was used to adjust the pension paid.^4^

**TABLE 1:** 2005 pension reform.

	Before reform	After reform
Full retirement age (FRA)	65	63
Early retirement age (ERA)	60	62
Early claiming penalty for each month	−0.4%	−0.6%
Reference age for early claimin	65	63
Delayed claiming bonus for each month	0.6%	0.4%
Reference age for delayed claiming	65	68
Accrual %		
Ages 18 to 22		1.5%
Ages 23 to 52	1.5%	1.5%
Ages 53 to 59	1.5%	1.9%
Ages 60 to 62	2.5%	1.9%
Ages 63 to 65	2.5%	4.5%
Ages 66 to 68		4.5%

*Note:* The reform maintained the eligibility age for the full national pension while adjusting the eligibility age for the early national pension from 60 to 62. There were no changes made to the penalty for claiming the national pension early.

The 2005 reform changed the fixed FRA to a flexible FRA. Since 2005 it has been possible to fully retire after the age of 63 and an individual's entire working history is taken into account in calculating the pension payments. The age limit of the ERA, on the other hand, was increased to 62. Early retirement cut pensions by 0.6% for each month of early retirement before the age of 63. Postponing retirement after the age of 68 increased pensions by 0.4% per month. In the reform, the eligibility age for the full national pension remained the same, but the eligibility age for the early national pension changed from 60 to 62. The penalty for claiming the national pension early did not change.

In addition to age limits and the associated rules, accrual rates changed as well. Following the reform, the accrual rate for the 18–52‐year‐olds was 1.5%. Between the ages 53 to 62, the accrual rate was 1.9%. For 63‐ to 68‐year‐olds, the reform introduced a so‐called “super” accrual rate of 4.5%, where the aim was to encourage people to keep working after the minimum eligibility retirement age. Further, pensions that accrued after the age of 63 no longer influenced an individual's national pension. The halfway index was replaced by a wage coefficient^5^ and the reform abolished the differences in the regulations between public and private sector workers.

## 3 Data and Empirical Strategy

### 3.1 Data and the Sample

We use individual‐level annual updated administrative data from the Finnish Centre for Pensions and Statistics Finland. In addition to crucial information on pensions and retirement decisions, the data includes a large set of individual demographic and labor market characteristics. In addition, the data contain a wide range of individual health indicators based on administrative data on hospital treatments, drug prescriptions and sickness absence. The sources for the health data are the Finnish Institute for Health and Welfare (THL) and the Social Insurance Institution of Finland (Kela). The sample is an 11% random sample of all persons residing in Finland for at least one year during 1987–2007 and the data for these individuals covers the years 2000–2009.

In the analysis, we concentrate on early and full old‐age retirement, and we include individuals entitled to earnings‐related pensions and/or national pensions. In Section 5, we check the robustness of our findings by focusing on individuals receiving only earnings‐related pensions, since they are the most affected by the changes in incentives caused by the reform.^6^

Our main sample includes private sector workers aged between 62 and 68 years (at the end of the year) who are in the labor force. Being in the labor force is defined as not having retired earlier. In one robustness check we run the analysis separately for individuals who were or were not unemployed in the previous year. We focus on 62‐ to 68‐year‐old individuals, because they are able to retire (early or full) both before and after the reform. All workers other than private sector workers are excluded from the sample, because of the inaccuracy of the data and some differences in the accrual rules between sectors. Our sample size is around 13,000 individual‐year observations.

We choose to exclude disability pensions from the main analysis since we want to focus on retirement that is based more directly on the decision of an individual. In Section 5, we examine the sensitivity of our results to the way in which disability pensions are handled, and show that our results are robust in this respect too.

As mentioned above, the reform took effect in 2005, but full information about the reform was available already in 2004. We exclude the years 2004 and 2005 from our analysis to abstract from potential anticipation effects.

### 3.2 Measuring the Financial Incentives to Retire

We use the changes in total pension wealth, when retirement is postponed by 1 year, to measure the financial incentives related to retirement. A similar approach has been used for example in Coile and Gruber (2007).^7^ We define pension wealth as the present value of the stream of future pension incomes until age one hundred. The benefit of using pension wealth instead of the annual pension is that it measures the financial incentives of postponing retirement more broadly. Analyzing changes in pension wealth takes into account how postponing retirement affects future pensions, as well as the fact that pension payments are then received for 1 year less. Formally, pension wealth is defined as:

(1) $$PW_{r}=\sum_{s=r}^{100}\pi_{s}\beta^{s-r}I^{s-r}P_{r}\left(r\right)$$

In Equation (1) r indicates the age of retirement, $P_{r}\left(r\right)$ is annual pension including national pension (in year 2000 euros) when retired at age r, s is age, $\pi_{s}$ represents gender and age dependent survival probability (from Statistics Finland) and β is the discount factor (β=0.97). After retirement, pensions are increased according to an index I in real terms.^8^ In the calculations of I we use the average annual growth rates of the consumer price index, earnings index and national pension index from 1995 to 2015.^9^

Our measure for financial incentives is the expected relative change in pension wealth when retirement is postponed by 1 year, based on the information available in period r:

(2) $$\Delta \;\ln\left(PW_{r}\right)=E_{r}\left[\ln\left(PW_{r+1}\right)\right]-\ln\left(PW_{r}\right)$$

We expect that when this measure increases, it becomes less likely that the individual retires within a year. The notion in Equation (2) is referred to as the “financial incentive to postpone retirement” in the remaining text, or sometimes simply “incentive” for brevity. We calculate pension wealth for each individual for every year using the accrual rules in place during a particular year. In the calculations, we use information about the individual's overall accrued pension at the end of 2004. The source for this variable is the Finnish Centre for Pensions. We further assume that the retirement date is always December 31. The main reason is that individuals who are not retired at all do not have any retirement date to use, and a fixed date within the year ensures comparability. Furthermore, for the retirement year and the year after retirement, we use earnings from the year before retirement multiplied by the earnings index.

**FIGURE 1:**
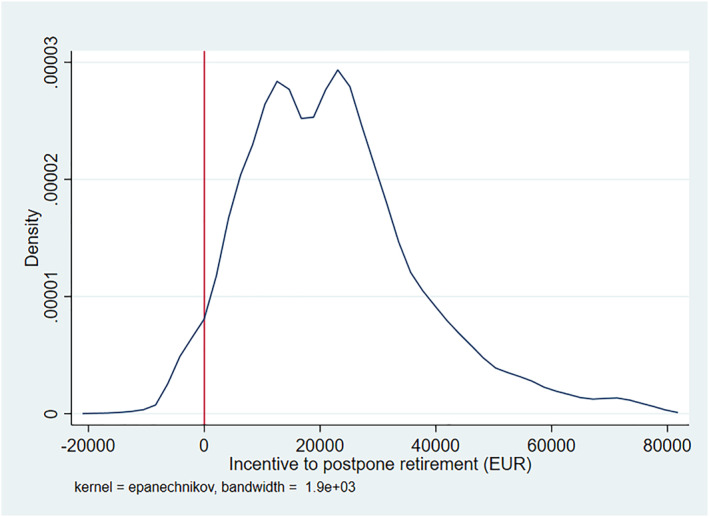
The distribution of retirement incentives (EUR). The Figure displays the distribution of the change in the amount of pension wealth when retirement is postponed by 1 year that is the financial incentive to postpone retirement, measured in euros. Pension wealth includes both earnings‐related pensions and national pensions. The values are in year 2000 euros and extreme values are excluded.

Figure 1 shows the distribution of the financial incentive to postpone retirement for different individuals. The Figure displays the financial incentives in absolute euro terms for clarity and transparency, while our econometric analysis below will utilize relative incentive measures (as defined in Equation 2).

The figure indicates that for the majority of individuals, postponing retirement increases their pension wealth and on average the increase is around 25,000 euros. However, the financial incentive to postpone retirement varies substantially among individuals. For some individuals, postponing retirement decreases pension wealth, whereas for some their pension wealth increases by over 80,000 euros.

To showcase how the incentives to postpone retirement were altered in the reform, we next present results from a simulation exercise. We first calculate pension wealth including both earnings‐related pension and national pension^10^ and the incentives to postpone retirement for every individual with both rules, and then compare these two. Since wealth and incentives are calculated for each individual under both rules for the whole study period, the only difference between the calculated values is due to the changes in the rules. Note that in the actual regression analysis, we do not only use a sample of the same individuals in a panel, rather our data are in a repeated cross‐section format.

**FIGURE 2:**
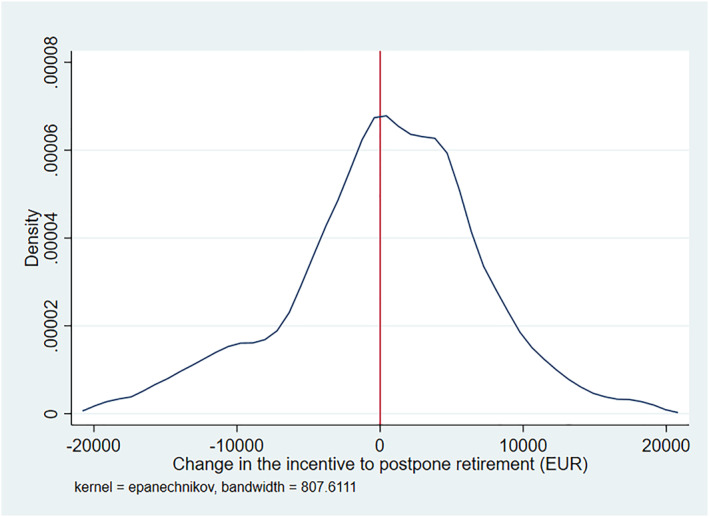
The distribution of the change in retirement incentives (EUR) in the 2005 reform. The figure displays the distribution of the change implied by the 2005 reform in the financial incentive to postpone retirement, measured in euros. Pension wealth includes both earnings‐related pensions and national pensions. The values are in year 2000 euros and extreme values are excluded.

Figure 2 illustrates how the reform changed the financial incentives to postpone retirement in euro terms. On average the change in financial incentives to postpone retirement is slightly negative, indicating that the reform on average mildly worsened the financial incentives to delay retirement. However, the implications for incentives are not the same for every individual and there is large variation as to how the reform changed incentives.

**FIGURE 3:**
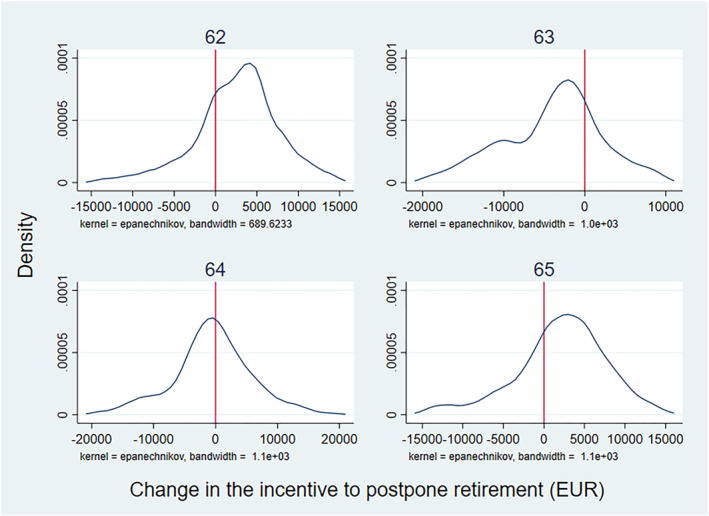
The distribution of the change in retirement incentives (EUR) in the 2005 reform, by age group. The figure displays the distribution of the change implied by the 2005 reform in the financial incentive to postpone retirement, measured in euros, by age group. Pension wealth includes both earnings‐related pensions and national pensions. The values are in year 2000 euros and extreme values are excluded.

As was explained above, different age groups were affected differently by the reform. Figure 3 shows how the reform changed incentives by age groups (also in euros). According to the Figure, the reform increased, on average, the incentives to postpone retirement for 62‐ and 65‐year‐old individuals. For 63‐ and 64‐year‐olds, the incentives worsened on average. In addition, the reform implied a direct, overnight, increase in pension wealth, driven by the reduction in the eligibility limit for full old‐age pension from 65 to 63 years of age. These changes are further described in Appendix B.

**FIGURE 4:**
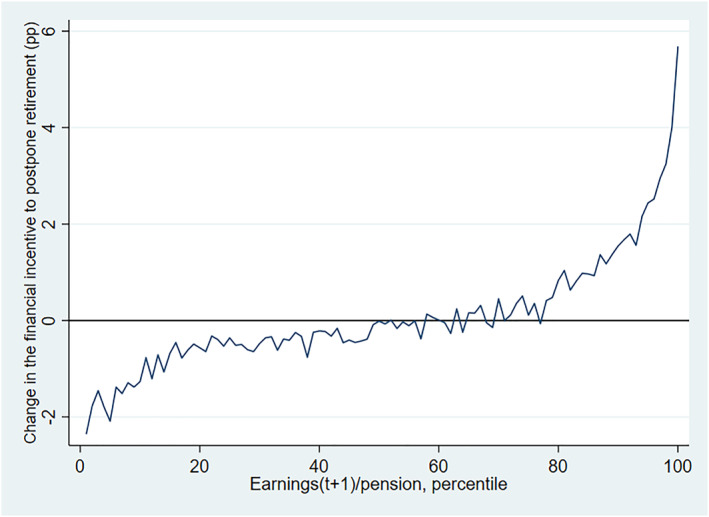
The change in retirement incentives in the 2005 reform, by the ratio of future earnings and pension. The figure displays the change in the financial incentive to postpone retirement implied by the 2005 reform, by the ratio of future earnings and pension. Pension wealth includes earnings‐related pensions and national pensions.

The reform created variation in incentives not only by cohort, but also according to accrued pension and the level of earnings. This variation in incentives is illustrated in Figure 4. On the *x*‐axis, individuals are divided into percentiles according to the ratio between earnings in the following year and current‐year total pension. The *y*‐axis shows the change in the financial incentive to delay retirement, implied by the reform. As can be seen from Figure 4, the incentives to postpone retirement improved for those with high earnings compared to their accrued pension and worsened for those with low earnings compared to their accrued pension. The change in financial incentives implied by the reform also clearly increases and is roughly monotonous over the percentiles, but the increase is not linear.

### 3.3 Measurement of Health

We measure health with several different variables, derived from various national registers containing detailed individual‐level information on different aspects of health.^11^

First, we use hospitalization and treatment information from the hospital discharge records of the Finnish Institute for Health and Welfare (THL). Using this register, we form an indicator of whether or not an individual has been treated in hospital for any reason. We also analyze cardiovascular diseases and diseases of the musculoskeletal system and connective tissue separately, with the identification of these conditions based on the International Classification of Diseases and Causes of Death (ICD‐10).^12^

Second, we use medication information from the reimbursement register of the Social Insurance Institution of Finland. We form two variables using data on purchases of prescription medications. The first measure is formed according to total purchases of any medication within a year and is divided into three categories (fewer than 4 purchases, 4 to 7, and 8 or more). The rationale for using the number of purchases as a proxy for health problems is the following: according to Finnish health insurance law, one can purchase 3 months worth of subscription medication, subject to reimbursement from public health insurance, at any one time. Therefore, four purchases within a year is a proxy for a treatment need of a chronic condition associated with particular medication. Further purchases signify that the individual is likely treated for multiple conditions or takes several different types of medication. The second measure relates specifically to mental health, where we use information on purchases of prescribed psychotropic medication to form an indicator of mental health problems.^13^

Third, we use information on sickness absence from the Social Insurance Institution of Finland. We measure sickness absence as the total length of all sickness absence spells in days within a year. Sickness allowances, however, are paid only after a specified (usually 10 days) waiting period and thus the register only contains information about those sickness absences that are longer than the waiting period. We divide the length of sickness absences (days after the waiting period) into four categories (0 days, 1–14 days, 15–60 days, and over 60 days).^14^

In addition to these measures, we form a more general indicator of ill health, dividing the sample into sub‐samples according to overall health status. We describe the formation and rationale behind this measure when we carry out the sub‐sample analysis.

A couple of notes on the nature of our health data are in order.

First, regarding sectoral coverage, the hospital discharge records relate to use of specialized health care, and these data have very good coverage of both publicly and privately provided overnight hospital care services. Coverage of outpatient care in specialized healthcare is limited, on the other hand. Therefore these data likely provide information on relatively severe cases of ill health. More information on the coverage of these data is provided in Sund (2012).^15^ However, our other health measures cover all sectors of health care provision and information on medication purchases is thus available regardless of where the medication was prescribed (primary or secondary care; and public, private, or occupational healthcare). In addition, information on sick leave does not hinge on where the leave was prescribed.

Second, regarding measurement of health using administrative data, the hospital discharge data and the prescription data cover only health problems for which individuals have sought treatment. This is a common feature when using administrative data to measure individual health. All purchases of prescription medication in Finland are from authorized pharmacies by prescription from a medical doctor after clinical assessment. Thus, our health measures, particularly those based on hospital use, are likely to capture more severe health problems.

Third, sickness absence is a particularly important health measure for our purposes, for two reasons: This variable covers cases where prior treatments—whether sought on time or not—have not been effective in maintaining the individual's working capabilities. Further, this measure directly captures the labor market‐relevant aspects of health, and is of particular interest when analyzing individual choices between work versus retirement. Indeed, as we show below, this is the health measure in our data that is most strongly correlated with the likelihood of retirement in a given year.

### 3.4 Descriptive Statistics

Table 2 shows some descriptive statistics for our main sample. The information is provided separately for the periods before and after the reform. While there are variables such as age and gender that are not expected to change over the period, many others are either directly or indirectly influenced by the reform, such as pension (wealth) levels. Also, general time trends may influence the health‐related variables.

**TABLE 2:** Means of selected variables before and after the reform.

	Before the reform	After the reform
Retirement rate	0.202	0.295
Reaching full retirement age	0.144	0.321
Female	0.430	0.395
Age (at the end of the year)	63.13	62.96
Spouse, share	0.706	0.715
Working history (years)	34.87	37.08
Unemployed, share (*t* − 1)	0.0504	0.0966
Tertiary degree, share	0.0998	0.137
Earnings	21,518	26,037
Accrued pension (euros)	13,132	16,851
Pension wealth (euros)	279,220	348,091
Pension wealth (logs)	12.43	12.63
Δln(PW)	0.0659	0.0678
Δ(PW)	20,379	24,855
Psychotropic medication (*t* − 1)	0.0681	0.0917
Medication purchases (*t* − 1)	5.688	7.022
Any treatment (*t* − 1)	0.106	0.114
Treatment, cardio (*t* − 1)	0.0228	0.0217
Treatment, muscular (*t* − 1)	0.0249	0.0236
Sickness absences days (*t* − 1)	5.170	4.909
Sickness absences share (*t* − 1)	0.127	0.115

*Note:* Monetary values are in year 2000 euros. Δln(PW) is the financial incentives to postpone retirement (i.e. the relative change in pension wealth, PW, when retirement is postponed by 1 year). Sickness absences in days refers to the length of sickness absence spells after the waiting period. The share of unemployed in the previous year is identified by using information about the main type of activity of an individual within a year.

Approximately 20%–30% of the target group retires each year, and a much larger share (32% instead of 14%) reaches the full retirement age after the reform, due to a lowering of the full retirement age. Individuals have approx. 35 years of work history on average, and the mean pension is close to 15,000 euros a year in comparison to mean annual earnings of around 24,600 euros. When it comes to the measures of health, a small share receive treatment for a specific type of illness. Many individuals (around 12%) have sickness absences and use a large amount of prescription medication. For example, 7%–9% of the individuals in the sample used psychotropic medication.

### 3.5 Estimation Strategy

Our main question is how financial incentives affect retirement behavior and whether the reactions vary between individuals with different health status. Our analysis includes the years around the 2005 reform: years 2001–2003 (before) and 2006–2008 (after the reform). The years 2004 and 2005 are dropped to abstract from possible anticipation effects related to the reform.

We exploit variation in the financial incentives to postpone retirement between population groups, created by the reform, to study how incentives affect retirement. To be precise, we estimate the following regression:

(3) $$R_{i,t}=\theta_{1}\Delta \;\ln\left(PW_{i,t}\right)+\theta_{2}\;\ln\left(PW_{i,t}\right)+\gamma_{t}+\beta X_{i,t}+u_{i,t}$$

where $R_{i,t}$ is an indicator variable equal to one if individual i retires in year t, conditional on not having retired earlier. The time fixed effect is denoted by γ. The vector X contains individual‐level control variables, such as gender, age, work history, and education, spouse controls and health indicators.^16^ $PW_{t}$ is an individual's calculated pension wealth at the end of year t and $\Delta \;\ln\left(PW_{i,t}\right)$ captures the incentives to postpone retirement.

The parameter of interest is $\theta_{1}$, which captures the effect of financial incentives on the retirement probability, while the control variables (including the actual level of pension wealth) capture some of the other determinants of retirement behavior.

To highlight the key features of our empirical strategy, our main specification resembles a difference‐in‐difference type analysis: We utilize data from years before and after the reform, and the reform caused financial incentives to change differently for different population groups. These changes were exogenous that is it is not possible for individuals to select into a particular group; we however note one potential caveat to this feature below. The regression includes controls for the relevant characteristics of those groups (similar to group dummies in a DiD analysis). Instead of having one treatment dummy, in our setting treatment intensity varies according to how much incentives changed in the reform.

After analyzing average responses, we study how the reactions to incentives vary by health status. We choose to present models estimated using different sub‐samples, because we favor an approach that allows the importance of other determinants of retirement decisions to vary across different population groups. This approach is more flexible and easier to interpret than the alternative of interacting the group indicators with the incentive measures. Nevertheless, we assess the robustness of our findings by also estimating a unified model that incorporates interaction terms between the incentives, pension wealth, reaching full retirement age, and the health variables.

As explained above, our main empirical strategy utilizes the pension reform as a source of exogenous variation in retirement incentives. Most individual characteristics that influence the financial incentives to postpone retirement and the way the reform changed those incentives (such as accrued pension, gender, and age) are exogenous near retirement. However, accrual rates and hence our measure of financial incentives depend on earnings, as shown in Figure 4. Earnings levels on the other hand may reflect changes in labor supply, or underlying differences in the preference for working, and this part of the variation in the incentive measure may be partially endogenous.

Therefore, as a robustness check, we complement our main analysis by implementing an IV analysis. Here, we follow an approach often used in estimating individual responses to tax changes, introduced by Gruber and Saez (2002). In order to isolate only the exogenous variation in incentives caused by the reform, the idea is to construct the incentive measure for each year using the accrual rules in place in that year, but calculating them with *lagged* earnings instead of current earnings.

We present the IV results only after the baseline OLS analyses as a robustness check. Reassuringly, the results are very similar, and the potential endogeneity therefore appears to play little role in our case.

It will be helpful to introduce some additional notation to explain the instrument used in more detail. The key regressor is the financial incentive to postpone retirement, $\Delta \;\ln\left(PW_{t}\right)=\ln\left(PW_{t+1}\right)-\ln\left(PW_{t}\right)$. Let us denote the log of the pension wealth by $\ln\left(PW_{t}\left(y_{t},\text{…}\right)\right)$, because the pension wealth depends, among other things, on the earnings level, y, and the pension wealth is calculated using the policy rules of year t.

Now the instrument for $\ln\left(PW_{t+1}\left(y_{t+1},\text{…}\right)\right)-\ln\left(PW_{t}\left(y_{t},\text{…}\right)\right)$ is $\ln\left(PW_{t+1}\left(y_{t},\text{…}\right)\right)-\ln\left(PW_{t}\left(y_{t-1},\text{…}\right)\right)$.^17^ The exclusion restriction is that individuals cannot affect previous earnings and therefore the only variation in the incentives come from the policy change, which is exogenous from the viewpoint of the individuals.

In addition to these main analyses, we carry out a wide variety of additional robustness checks, as outlined in Section 5.

## 4 Results

### 4.1 Results for the Full Sample

**FIGURE 5:**
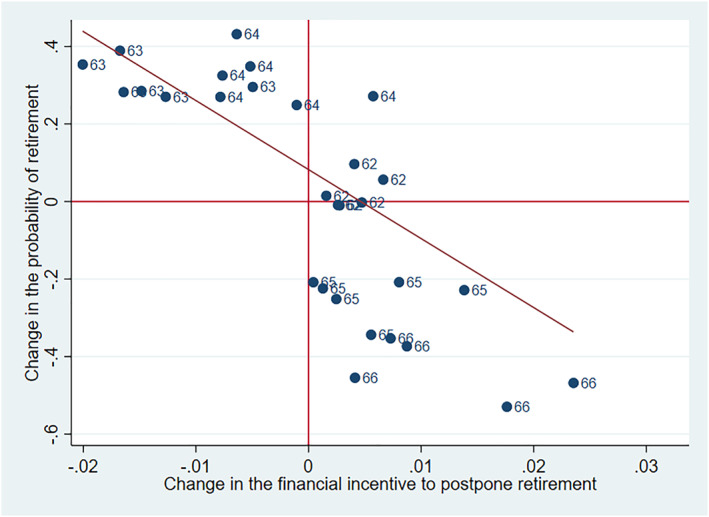
Relationship between the change in retirement probability and the change in retirement incentives in the 2005 reform. The figure displays the relationship between the change in the financial incentive to postpone retirement (*x*‐axis) implied by the 2005 reform, and the change in retirement probability (*y*‐axis), for different groups. The groups are formed according to age, gender, and length of work history (three categories). The displayed age represents the age of the group at the end of the year. The red line is the regression line, which is weighted by group size.

We first provide some graphical evidence to illustrate how changes in the financial incentives to postpone retirement, due to the reform, are related to the corresponding change in the probability of retirement. This is depicted in Figure 5. The *x*‐axis shows how the reform changed the incentives in relative terms among various groups, whereas the *y*‐axis shows how the probability of retirement changed in the same groups around the reform. The groups are formed according to age, gender and work history (less than 35 years, 35–40 years, at least 40 years) and the numbers in the graph show the age of each group. Clearly there is a negative connection between the incentives to postpone retirement and the retirement probability.

Next, we report the mean impact of the reform on retirement using the specification in Equation (3). These results are shown in Table 3, where we successively add more control variables in each column. The first column reports results from the regression with year fixed effects, while the second column adds the level of pension wealth to the model. The third model contains basic individual‐specific control variables, which include also a dummy measuring whether the person has reached the full retirement age.^18^ Controlling for this factor is important, given that reaching the default retirement age has been found to be a crucial determinant of retirement decisions (Gruber, Kanninen, and Ravaska 2022). The fourth model also includes controls for an individual's spouse. In models (5) to (10), the health variables are included separately one at a time, and in model (11) all the individual health variables are included simultaneously.

**TABLE 3:** Baseline OLS estimates.

	(1)	(2)	(3)	(4)	(5)	(6)	(7)	(8)	(9)	(10)	(11)
Dependent variable is the retirement decision and mean retirement rate in each model is 0.265											
Δln(PW)	−3.929***	−4.157***	−2.216***	−2.227***	−2.226***	−2.229***	−2.227***	−2.225***	−2.225***	−2.243***	−2.244***
	(0.126)	(0.129)	(0.126)	(0.126)	(0.126)	(0.126)	(0.126)	(0.126)	(0.126)	(0.126)	(0.126)
ln(PW)		0.103***	0.0489***	0.0482***	0.0481***	0.0482***	0.0482***	0.0483***	0.0479***	0.0481***	0.0480***
		(0.00816)	(0.0108)	(0.0108)	(0.0108)	(0.0108)	(0.0108)	(0.0108)	(0.0108)	(0.0108)	(0.0108)
FRA			0.238***	0.237***	0.237***	0.237***	0.237***	0.237***	0.237***	0.237***	0.238***
			(0.0127)	(0.0127)	(0.0127)	(0.0127)	(0.0127)	(0.0127)	(0.0127)	(0.0127)	(0.0127)
Treatment, cardio (*t* − 1)					0.0170						0.0109
					(0.0228)						(0.0256)
Treatment, muscular (*t* − 1)						0.0356					0.0246
						(0.0223)					(0.0254)
Any treatment (*t* − 1)							0.00794				−0.0223
							(0.0105)				(0.0139)
Psychotropic medication (*t* − 1)								0.0413***			0.0324*
								(0.0125)			(0.0128)
Medication purchases (t−1)											
4 to 7									−0.00270		−0.00565
									(0.00852)		(0.00855)
8 or more									0.0219**		0.0139
									(0.00755)		(0.00786)
Sickness absences (t−1)											
1–14 days										0.0330*	0.0335*
										(0.0160)	(0.0165)
15–60 days										0.0195	0.0217
										(0.0148)	(0.0159)
Over 60 days										0.0730***	0.0697**
										(0.0221)	(0.0237)
Observations	12,977	12,977	12,923	12,923	12,923	12,923	12,923	12,923	12,923	12,923	12,923
*R*‐squared	0.087	0.099	0.318	0.319	0.319	0.319	0.319	0.320	0.320	0.320	0.321
FE	YES	YES	YES	YES	YES	YES	YES	YES	YES	YES	YES
Individual controls	NO	NO	YES	YES	YES	YES	YES	YES	YES	YES	YES
Spouse controls	NO	NO	NO	YES	YES	YES	YES	YES	YES	YES	YES
Health controls *t* − 1	NO	NO	NO	NO	NO	NO	NO	NO	NO	NO	YES

*Note:* Δln(PW) is the financial incentives to postpone retirement (i.e. the relative change in pension wealth, PW, when retirement is postponed by 1 year). Sickness absences indicates days after the waiting period. Robust standard errors in parentheses ***p < 0.001, **p < 0.01, *p < 0.05.

The signs of the estimates are as expected. The coefficient of the log change in pension wealth, Δln(PW), is negative, indicating that when financial incentives to postpone retirement are increased, individuals are indeed less likely to retire during the analysis year. On the other hand, the level of accrued pension wealth itself (ln(PW)) is associated with a higher likelihood of retirement. These results are stable across the different specifications. With the full set of controls (Column 11), the estimated coefficient indicates that a 1% point increase in the financial incentives decreases the risk of retirement by around 2.2% points. A one‐unit increase in the level of log of pension wealth, in turn, increases the risk of retirement by 4.8% points (Column 11). Compared to earlier findings from Finland (Uusitalo and Nivalainen 2013), our estimation results for the effect of incentives have the same sign, but the magnitude is smaller, which may be due for example to the fact that Uusitalo and Nivalainen (2013) conduct the analysis at a group level. In Sweden, Johansson, Laun, and Palme (2014) find rather similar results on the effects of incentives as we do. In sum, individuals respond to retirement incentives as expected, and the positive association between the level of wealth and retirement is consistent with the notion that leisure time is a normal good (higher wealth levels lead to earlier retirement).

As in Gruber, Kanninen, and Ravaska (2022), we find that reaching the full retirement age (FRA) is an important determinant of the retirement decision. The estimated coefficient on the dummy indicating that an individual has reached the statutory retirement age is around 0.24 across all specifications.

Regarding the health variables, in many cases worse health is associated with a higher risk of retirement. Psychotropic medication increases the risk of retirement by 4.1% points and having at least 8 prescription medication purchases increases it by around 2.2% points. Sickness absence increases the risk of retirement as well. Having a spell of 1–14 sickness absence days (after the waiting period) increases the risk by around 3.3% points and over 60 sickness days increases it by around 7.3% points.^19^ When the health variables are included simultaneously, the use of psychotropic medication and having had 1 to 14 or at least 60 sickness absence days remain statistically significant determinants of retirement behavior. In particular, long spells of sickness absence are, quite intuitively, important determinants of retirement behavior.

### 4.2 Results by Subgroups

Next, we analyze how the results vary between different types of individuals with varying health. The heterogeneity analyses are conducted by running regressions using separate sub‐samples. The samples are divided according to whether an individual has (i) had any treatment in a hospital; (ii) received treatment for cardiovascular diseases or (iii) musculo‐skeletal diseases; (iv) had 8 or more purchases of medication; (v) had medication for mental illnesses; and (vi) had at least one sickness absence day after the waiting period.

Finally, we form an indicator of ill health which combines information from the different health measures. This combined measure is a complement to the individual measures, and attempts to capture the individuals' health status more broadly. The indicator gets value one if the individual has been an inpatient due to cardiovascular diseases in a particular year, has 8 or more purchases of prescription medication, has psychotropic medication or has over 60 days of sickness absence. The rationale behind this indicator is to make the two groups—more and less healthy persons—more equal in terms of their size.

Our interest here is in the equity effects of the reform, in particular the interaction between health inequality and economic inequality. We would like to examine whether the reform has a differential effect on those who are prone to retire earlier due to health problems in the baseline. Do those individuals who have a larger risk of retiring early react to incentives ‐ and correspondingly, are they able to utilize the potential financial benefits created by the reform? It is possible, of course, that the individuals differ in other respects than health as well, but we attempt to mitigate this issue by always including a large number of control variables (also related to the spouse).

The purpose of the subgroup analysis is therefore to detect whether there is a risk that providing incentives for continuing to work have ramifications in terms of aggravated inequality among older workers. However, it should also be noted that it is not necessarily clear that those with worse health are less inclined to react to incentives: it is also possible that they in fact require stronger financial returns for continuing to work to compensate for the greater opportunity cost of working. The pattern of heterogeneity that we should expect to see is therefore not clear a priori, making the empirical subgroup analysis all the more important and interesting. Further, the link between health and the reaction to incentives may of course differ depending on the type of the underlying health issues.

The results for the subgroup analysis, using our main specification corresponding to Equation (3) with the full set of controls, are reported in Table 4. Overall, those with different types of health problems mostly do react to the incentives and to the level of pension wealth in a similar way as individuals in our sample overall. This is revealed by the test statistics indicating whether the estimated coefficient of the financial incentives differ in a statistically significant way between the two groups studied. This holds for most health indicators. An important finding, therefore, is that many types of individuals do appear to react to retirement incentives.

**TABLE 4:** OLS estimates by population groups.

	Treatment, cardio (*t* − 1)		Treatment, muscular (*t* − 1)		Any treatment (*t* − 1)		Medication (*t* − 1)		Psychotropic medication (*t* − 1)		Sickness absences (*t* − 1)		Ill health (*t* − 1)	
	Yes	No	Yes	No	Yes	No	8≤	7 or less	Yes	No	1≤	No	Yes	No
Dependent variable is retirement decision														
Δln(PW)	−3.108**	−2.207***	−2.843***	−2.220***	−2.363***	−2.225***	−2.153***	−2.267***	−2.342***	−2.218***	−1.105**	−2.352***	−2.293***	−2.190***
	(1.001)	(0.127)	(0.850)	(0.128)	(0.365)	(0.134)	(0.208)	(0.159)	(0.399)	(0.133)	(0.418)	(0.132)	(0.195)	(0.166)
ln(PW)	−0.126	0.0506***	0.0511	0.0485***	0.0254	0.0516***	0.0528**	0.0426**	0.0613	0.0471***	0.0422	0.0493***	0.0516**	0.0432**
	(0.0805)	(0.0110)	(0.0937)	(0.0109)	(0.0342)	(0.0114)	(0.0183)	(0.0135)	(0.0378)	(0.0113)	(0.0349)	(0.0114)	(0.0175)	(0.0139)
FRA	0.253*	0.237***	0.0453	0.240***	0.185***	0.242***	0.254***	0.227***	0.219***	0.237***	0.156***	0.248***	0.253***	0.227***
	(0.101)	(0.0129)	(0.114)	(0.0128)	(0.0423)	(0.0134)	(0.0219)	(0.0157)	(0.0476)	(0.0133)	(0.0378)	(0.0135)	(0.0206)	(0.0162)
*p*‐value Δln(PW)	0.321		0.427		0.718		0.662		0.761		0.004		0.685	
*p*‐value ln(PW)	0.016		0.975		0.459		0.652		0.712		0.845		0.707	
*p*‐value FRA	0.861		0.06		0.191		0.303		0.705		0.02		0.307	
Observations	287	12,636	313	12,610	1447	11,476	4501	8422	1088	11,835	1543	11,380	4990	7933
R‐squared	0.438	0.319	0.412	0.320	0.320	0.322	0.312	0.327	0.312	0.324	0.343	0.320	0.313	0.327
FE	YES	YES	YES	YES	YES	YES	YES	YES	YES	YES	YES	YES	YES	YES
Individual controls	YES	YES	YES	YES	YES	YES	YES	YES	YES	YES	YES	YES	YES	YES
Spouse controls	YES	YES	YES	YES	YES	YES	YES	YES	YES	YES	YES	YES	YES	YES
Health controls t‐1	NO	NO	NO	NO	NO	NO	NO	NO	NO	NO	NO	NO	NO	NO

*Note:* The null hypothesis for the *p*‐values is that the difference between the coefficients equals zero. Δln(PW) is the financial incentives to postpone retirement (i.e. the relative change in pension wealth, PW, when retirement is postponed by 1 year). Sickness absences indicates days after the waiting period. Robust standard errors in parentheses ***p < 0.001, **p < 0.01, *p < 0.05.

However, the reactions of individuals with sickness absence spells (exceeding the 10‐day deductible period) differ from those of other individuals in our sample: individuals with an extended period of sickness absence react less to the financial incentives to postpone retirement than others do. Furthermore, they react less to reaching the full retirement age. The finding that we see differential reactions for this group in particular is of interest, given that sickness absence is a health indicator with the clearest a priori link to labor‐market behavior. Having an extended period of sickness absence also points to untreated or unmanaged health problems, as we discussed in Section 3.3. The information provided in Table A3 sheds light on the issue how individual characteristics differ between those with or without sickness absence. People with sickness absence have, indeed, much more health issues than the persons without sickness absence also according to the other health indicators. For instance, those with sickness absence have had 10 times more often at least some treatment in the previous year. The sickness absence variable may thus best capture the least healthy group of people. These individuals are also less well off than others for example they are less well educated and have a lower level of accrued pension wealth.

To summarize, the results indicate that economic incentives and the level of pension wealth as well as health status matter for retirement decisions, and many types of individuals, with different health status, do appear to react to incentives. However, since those with sickness absence react less strongly to economic incentives, this group may need special attention when planning pension policies. For instance, some individuals in this group might benefit from similar treatment as those actually covered by disability insurance; in other words, access to retirement before the rest of the population.

An interesting question relates to the comparison of our results with those of Leinonen et al. (2016), who found that relatively healthier individuals started to retire earlier after the reform. This finding is not inconsistent with our results. First, one should check whether the reform changed incentives differently according to health. However, the figures in Appendix: The Effect of the Reform on Incentives According to Health Statuses indicate no systematic differences in this respect. One potential explanation for the finding in Leinonen et al. (2016), which is consistent with what we find, is that according to our results healthy individuals appear to react somewhat more strongly to the statutory full retirement age, which also changed at the reform (c.f., Table 4). While it is difficult to pinpoint the precise reason behind this difference, one potential explanation is that less healthy individuals are more likely to have already retired before reaching the full retirement age.

## 5 Robustness

### 5.1 Earnings‐Related Pensions

In the analysis above we included individuals receiving earnings‐related pension and/or national pension. However, the reform mainly concerned the earnings‐related pension system, and individuals receiving only earnings‐related pension were affected more than individuals receiving national pension. In addition, individuals receiving only earnings‐related pension have overall higher levels of pension wealth. These two observations together indicate that there might be differences in responses between these two types of individuals.

For this reason, we also examined the recipients of earnings‐related pension only, excluding those individuals who received any amount of national pension. The results of the full model (corresponding to the specification reported in the last column of Table 3 for the full sample) are reported in the first column of Table 5. The impact of financial incentives to postpone retirement remains significant for this smaller sample as well, but the magnitude of the coefficient rises. This is to be expected, since the changes in the incentives are muted for those receiving national pension.

**TABLE 5:** Robustness of the baseline results.

	OLS						2SLS	
	(1)	(2)	(3)	(4)	(5)	(6)	(7)	(8)
Dependent variable is retirement decision								
Δln(PW)	−2.680***	−1.335***	−3.730***	−2.174***	−2.305***		−2.380***	−2.864***
	(0.176)	(0.137)	(0.598)	(0.128)	(0.125)		(0.129)	(0.146)
ln(PW)	0.0628***	0.0422***	0.184***	0.0466***	0.0500***		0.049***	0.046***
	(0.0158)	(0.0112)	(0.0407)	(0.0110)	(0.0107)		(0.011)	(0.011)
Δln(pension)						−2.243***		
						(0.126)		
ln(pension)						0.0488***		
						(0.0109)		
FRA	0.253***	0.241***	0.199***	0.239***	0.237***	0.238***	0.237***	0.239***
	(0.0163)	(0.0133)	(0.0385)	(0.0130)	(0.0127)	(0.0127)	(0.013)	(0.014)
Treatment, cardio (*t* − 1)	−0.00189	0.00479	0.0272	0.00557	0.0110	0.0108	0.011	0.016
	(0.0322)	(0.0267)	(0.0880)	(0.0267)	(0.0255)	(0.0256)	(0.025)	(0.027)
Treatment, muscular (*t* − 1)	0.0376	0.0263	−0.0165	−0.0120	0.0241	0.0246	0.025	0.038
	(0.0331)	(0.0261)	(0.112)	(0.0266)	(0.0253)	(0.0254)	(0.025)	(0.027)
Any treatment (*t* − 1)	−0.0240	−0.0207	−0.0614	−0.0378**	−0.0224	−0.0223	−0.023	−0.034*
	(0.0181)	(0.0145)	(0.0447)	(0.0146)	(0.0139)	(0.0139)	(0.014)	(0.014)
Psychotropic medication (*t* − 1)	0.0340*	0.0326*	−0.00616	0.0470***	0.0325*	0.0324*	0.032*	0.031*
	(0.0167)	(0.0135)	(0.0380)	(0.0132)	(0.0128)	(0.0128)	(0.013)	(0.013)
Medication purchases (*t* − 1)								
4 to 7	−0.0113	−0.00580	0.0306	−0.00545	−0.00599	−0.00566	−0.006	−0.008
	(0.0107)	(0.00875)	(0.0325)	(0.00876)	(0.00854)	(0.00855)	(0.009)	(0.009)
8 or more	0.0188	0.0100	0.0463	0.0197*	0.0134	0.0139	0.014	0.016
	(0.00987)	(0.00805)	(0.0293)	(0.00806)	(0.00784)	(0.00786)	(0.008)	(0.008)
Sickness absenses (*t* − 1)								
1–14 days	0.0354	0.0415*	−0.0137	0.0524**	0.0337*	0.0335*	0.034*	0.042*
	(0.0203)	(0.0166)	(0.104)	(0.0172)	(0.0165)	(0.0165)	(0.016)	(0.018)
15–60 days	0.0196	0.0303	−0.0747	0.0850***	0.0224	0.0217	0.023	0.030
	(0.0210)	(0.0164)	(0.0585)	(0.0175)	(0.0159)	(0.0159)	(0.016)	(0.017)
Over 60 days	0.0821**	0.0863***	−0.0571	0.361***	0.0685**	0.0697**	0.070**	0.073**
	(0.0315)	(0.0243)	(0.115)	(0.0237)	(0.0237)	(0.0237)	(0.024)	(0.025)
Mean retirement rate	0.274	0.248	0.459	0.283	0.265	0.265	0.265	0.273
Observations	8376	11,862	1061	13,244	12,923	12,923	12,923	11,712
R‐squared	0.326	0.316	0.449	0.297	0.323	0.321	0.321	0.306
FE	YES	YES	YES	YES	YES	YES	YES	YES
Individual controls	YES	YES	YES	YES	YES	YES	YES	YES
Spouse controls	YES	YES	YES	YES	YES	YES	YES	YES
Health controls *t* − 1	YES	YES	YES	YES	YES	YES	YES	YES
First stage: Dependent variable is the relative change in pension wealth calculated by lagged (one or 2 year) income								
Kleibergen‐Paap *F* statistics							58,220	12,248
First stage r‐squared							0.227	0.223

*Note:* In model (1), individuals entitled to national pension are excluded, in model (2) unemployed in the previous year are excluded, in model (3) only the unemployed in the previous year are included, in model (4) disability retirements are included and pension wealth and the incentives calculated according to Equation (4), in model (5) pension wealth is calculated using lagged income for all, in model (6) Δln(pension) and ln(pension), are used to measure the incentives. OLS is used for the first 6 models, and in all models except model (6) incentives are measured using the relative change in pension wealth and the level of pension wealth. In models (7) and (8) the estimation is conducted using 2SLS with the main sample, but year 2001 is excluded from model (8). In model (7) the relative change in pension wealth calculated by 1 year lagged earnings $\left(\ln\left(PW_{t+1}\left(y_{t},\text{…}\right)\right)-\ln\left(PW_{t}\left(y_{t-1},\text{…}\right)\right)\right)$ are used as instruments. In model (8) the relative change in pension wealth calculated by 2 year lagged earnings $\left(\ln\left(PW_{t+1}\left(y_{t-1},\text{…}\right)\right)-\ln\left(PW_{t}\left(y_{t-2},\text{…}\right)\right)\right)$ are used as instruments. Δln(PW) is the financial incentives to postpone retirement (i.e. the relative change in pension wealth, PW, when retirement is postponed by 1 year). FRA is an indicator for reaching full retirement age. Sickness absences indicates days after the waiting period. Robust standard errors in parentheses ***p < 0.001, **p < 0.01, *p < 0.05.

We also conduct the robustness analysis by partitioning the data into subgroups (see Table A6). The results tell a similar story as the main analysis. Most subgroups' retirement decisions react to the financial incentives to postpone retirement. Furthermore, individuals with sickness absence react less to incentives than other individuals.

### 5.2 Unemployed Individuals

Unemployed individuals may have different reasons to retire than employed individuals simply because they do not have a job to continue in. Furthermore, there might be differences in the usage of sickness absence between employed and unemployed individuals.^20^ Therefore we also run the analysis excluding individuals who were unemployed in the previous year from the sample. We have information about the main type of activity of an individual within a year and use that to identify unemployed individuals.

The baseline estimation results are shown in the second column of Table 5. The estimated coefficient for the effect of the incentives is much smaller than with the main specification, but still highly significant. As excepted, having at least one sickness benefit spell has a larger impact on the risk of retirement in this sub‐sample. On the other hand, if one only includes the unemployed (the third column in Table 5), the point estimate of the incentives strongly increase. The unemployed are therefore a group for whom the financial incentives to postpone retirement matter a great deal. A potential explanation is that a key difference between being unemployed versus retired is indeed the type of monetary compensation or social benefit received, rather than the amount of labor supply. Therefore other reasons that affect the labor supply versus retirement decision are likely less relevant for this subgroup, leading to a more pronounced role of monetary incentives.

The population subgroup results for the subgroup that excludes the unemployed are displayed in Table A7. The results are fairly similar to our main analysis, in that different types of individuals react to incentives to postpone retirement. In this subgroup, differences in reactions to incentives between individuals with and without a previous spell of sickness absence are qualitatively similar to the main analysis, but the difference is not statistically significant.

### 5.3 Disability Pensions

In the main analysis, we excluded disability pensions because we wanted to concentrate on retirement that is more directly based on the active decision of an individual. This choice may not necessarily be entirely innocuous. We are interested in individuals with different health statuses, and one may worry whether excluding individuals with the poorest health affects our results. In this section, however, we argue that the reliability of our results is not compromised by this choice.

First, Table 6 shows that the majority of disability retirements occur before the age of 62 and this share has remained similar after the reform. However, an additional worry is that the reform might have affected the attractiveness of the different retirement pathways. The change in the full retirement age also affected the eligibility ages for disability pensions: after the reform, 63‐ to 65‐year‐old individuals could no longer retire due to disability but were able to claim full old‐age pension. The reform also decreased the target age of the projected pension component and changed the accrual rate of the projected pension component from 0.8% to 1.3% and after 2010 it was further increased to 1.5%. Disability pensions were calculated with the new rules for the first time in 2006. In addition, the reform abolished the unemployment pension and this was partly compensated by providing occupational disability criteria for disability retirement.

**TABLE 6:** Share of retirements by age and retirement type.

Age	2003	2004	2005	2006	2007	2008
Old‐age retirements						
62	5%	4%	7%	9%	13%	13%
63	15%	17%	35%	36%	42%	44%
64	2%	5%	17%	11%	10%	10%
65	44%	44%	24%	23%	17%	18%
66	1%	1%	1%	1%	1%	2%
67	0%	0%	0%	0%	1%	1%
68	0%	0%	0%	0%	0%	1%
Total number of retirements	19,768	21,909	31,331	27,733	28,772	35,768
Retirement due to disability						
62	4%	3%	3%	3%	3%	4%
63	2%	2%	2%	1%	1%	1%
64	1%	1%	1%	0%	0%	0%
65	0%	0%	0%	0%	0%	0%
Total number of retirements	28,056	28,386	28,746	27,215	28,187	28,665

*Note:* Values include all working sectors. Shares are calculated relative to the total number of retirements of each type and year. Age is at the end of the year.

*Source:* Own calculations based on Finnish Centre for Pensions online database.

To account for the role of disability pensions, we carry out a robustness check where we include individuals over the age of 62 who retired due to disability in our sample. This increases the sample size by around 300 observations. However, there is a possible endogeneity problem related to the assignment of different retirement pathways, since the disability pensions are higher and the incentives to postpone retirement are poorer compared to old‐age retirement. Therefore, if we were to calculate retirement incentives according to the rules for disability pension, for all individuals who retired due to disability, we would likely end up overestimating the effect of incentives and underestimating the role of pension wealth.

To deal with this potential endogeneity issue, we follow the example of Johansson, Laun, and Palme (2014) and use a probabilistic approach to weight different pathways to retirement. We first calculate the share of individuals aged 62 to 65^21^ retiring through old‐age‐retirement or disability retirement by gender, education and year conditional on not having retired earlier. For this purpose we extended the sample to cover all working sectors (excluding individuals with personal retirement ages), and the descriptive statistics on the shares are shown in the Appendix D, Tables A4 and A5. Then these shares are used to form a weighted sum of pension wealth:

(4) $$PW\left(weighted\right)=\frac{p\left(DI\right)}{p\left(DI\right)+p\left(OLD\right)}PW\left(DI\right)+\frac{p\left(OLD\right)}{p\left(DI\right)+p\left(OLD\right)}PW$$

Where p(DI) is the mean disability retirement rate and p(OLD) is the mean old‐age retirement rate. As mentioned earlier, these rates vary according to gender, age and year. PW is pension wealth with old‐age pensions and PW(DI) is pension wealth with disability pensions. This weighted pension wealth is used to form the incentives to postpone retirement, when the possibility to retire due to disability is taken into account.

The baseline estimation results are displayed in the fourth column of Table 5 and the sub‐group results are shown in the Appendix, Table A9. Not surprisingly, the health variables are much more important determinants of retirement when disability retirement is taken into account. In particular, having a large number of sickness absence days increases the likelihood of retirement substantially (around 36 pp.). Overall the coefficient for the incentives to postpone retirement are almost the same as in the baseline estimation. The results of the sub‐group analysis are qualitatively similar to our baseline results, but differences in reactions to incentives between individuals with and without a previous spell of sickness absence are not statistically significant in this analysis.

### 5.4 IV Estimations

As explained in Section 3.5, we check the robustness of our baseline results using a complementary estimation strategy building on an IV approach.

In the first stage of the IV analysis, the dependent variable is the change in pension wealth, Δln(PW), which is regressed on the Gruber‐Saez (2002) type instrument, constructed using lagged income but current year's policy rules. The results regarding the mean impacts are shown in Columns 7 (using the instrument with 1 year lagged incomes) and 8 (2 year lagged incomes) of Table 5. The first‐stage *F*‐test result is very strong, confirming instrument strength. The results are qualitatively very similar to the OLS results ‐ the signs of the main coefficients of interest are as expected and the estimated coefficients are also statistically highly significant. This indicates that the potential endogeneity in the incentive measure did not turn out to plague our baseline OLS analysis. This increases our confidence that we have indeed isolated a causal relationship between the financial incentives to postpone retirement and the retirement decision.

Tables A10 and A11 show the 2SLS estimation results for the different population groups. Qualitatively, the picture that emerges is similar to the main analysis. Again, the differences in the reactions across population groups are typically not statistically significant. Only individuals with a sickness absence spell seem to differ in their reactions as they react less than those without a sickness absence spell. The sub‐group IV results are, therefore, similar to the corresponding OLS results.

### 5.5 Additional Robustness Checks

In columns (5) and (6) of Table 5 and in Appendix B, we present two additional robustness checks, dealing with changes in measuring incentives and using interaction terms, instead of split‐sample analysis, to examine heterogeneity in responses. Choosing to use the annual change in pension, rather than pension wealth, as an incentive measure leads to very similar results. When using interaction terms, the only statistically significant difference in the response pertains to sick leave, further confirming the results discussed above.

## 6 Conclusions

We analyzed the effects of retirement incentives on retirement behavior, using the Finnish pension reform implemented in 2005. The reform changed the financial incentives to postpone retirement differently for people with different labor market histories, and we exploit the exogenous variation in retirement incentives generated by the reform. The results indicate that the changes in financial incentives to postpone retirement influenced retirement decisions in an expected manner on average. Improved incentives to continue working induced individuals to postpone retirement, while a higher level of accrued pension wealth led to earlier retirement.

Our second key aim was to study how the reactions to incentives vary among different types of people. In particular, we analyzed how health status, measured using rich administrative data, may modify the effect of financial incentives on actual retirement decisions: Are all individuals, also those with ill health, able to take advantage of the monetary incentives to postpone retirement? On the one hand, older workers who have health problems may find it difficult to postpone retirement despite the financial gain of working longer. On the other hand, the financial gain may be especially important for those with worse health, as greater financial compensation is needed to offset the (mental or physical) costs of working. Depending on which mechanism dominates, ill health may lead to a muted or a more pronounced reaction to changes in incentives. To the best of our knowledge, similar analysis combining quasi‐experimental variation in retirement incentives, and focusing on this type of potential heterogeneity in the responses to these incentives, has not been offered in previous literature. While we implement this research in the Finnish context, we argue that the results have external validity, for example because of the objective health measures used, and because the types of incentives we examine are increasingly typical in pension reforms implemented in high‐income countries.

Using a wide array of health indicators, including inpatient hospital care and purchases of prescribed medication, we found that individuals with various different types of health issues do respond to incentives to postpone retirement. There are reasons to interpret these findings with some caution, as the health measures used might still hide some heterogeneity between individuals in the severity of morbidity.

An interesting exception to the general pattern highlighted above was that those with a sickness absence period exceeding 10 days—a measure of ill health specifically related to the ability to work—reacted less strongly than others to financial incentives to continue working. The individuals in this group also have many more health issues as indicated by the other health measures used, which suggests that the group has particularly severe conditions affecting functional ability. Sickness absenteeism further suggests that prior treatment of these conditions has not been effective in maintaining the individuals work capacity. The statistical significance of these results varies somewhat, however, depending on the sample used.

Overall, our results suggest that many different types of individuals, who receive treatment for different health conditions, are able to take advantage of financial incentives inherent in the pension system. However, individuals whose health problems have led to reduced working capabilities may not respond to financial incentives to postpone retirement in the same way as their more healthy peers. The associated equity/efficiency trade‐off may be mitigated by ensuring that there are suitable mechanisms in place, via for example disability insurance, for those who truly cannot continue working.

## Conflicts of Interest

The authors declare no conflicts of interest.

## Figures and Tables

**FIGURE A1:**
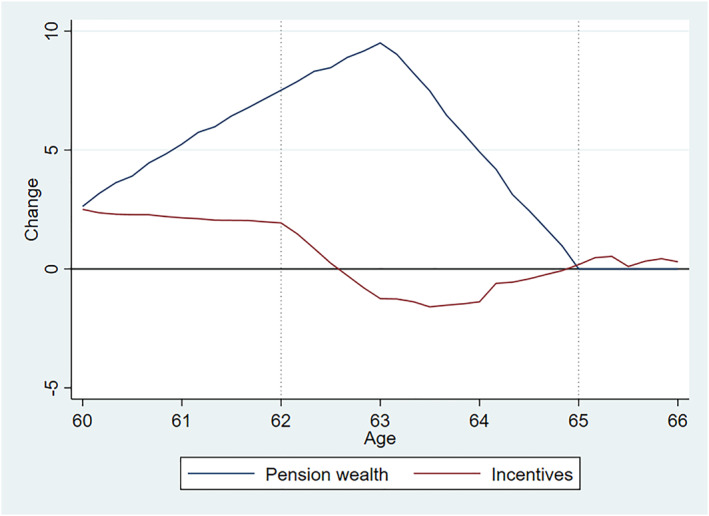
Changes in pension wealth and incentives to postpone retirement in the 2005 reform. The values are calculated by comparing the levels of pension wealth using the retirement rules before and after the reform. These are calculated as averages within bimonthly bins. The red line is calculated as a percentage point difference in the relative change in pension wealth when retirement is postponed by 12 months that is the line depicts the change in the financial incentive to postpone retirement, as defined in Equation (2). The blue line is calculated as a percentage change in pension wealth. The change in pension wealth is calculated using the information on total accrued pension at the end of year 2004 and using the 2004 data. Incentives are calculated using the whole sample. Age is age at the end of the year.

**FIGURE A2:**
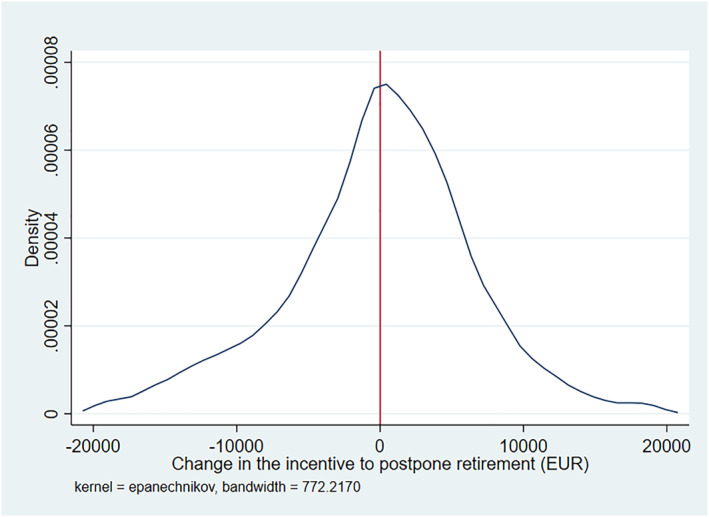
The distribution of the change in retirement incentives without national pensions (EUR) in the 2005 reform. The figure displays the distribution of the change implied by the 2005 reform in the financial incentive to postpone retirement, measured in euros. Pension wealth includes only earnings‐related pensions. The values are in year 2000 euros and extreme values are excluded.

**FIGURE A3:**
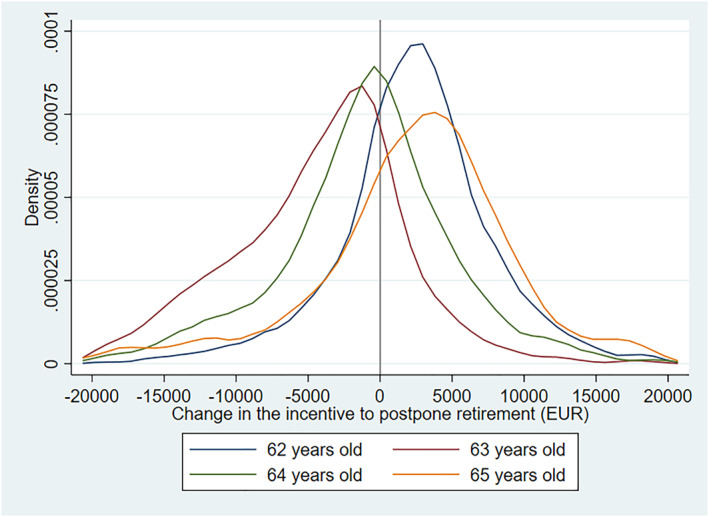
The distribution of the change in retirement incentives without national pensions (EUR) in the 2005 reform by age groups. The figure displays the distribution of the change implied by the 2005 reform in the financial incentive to postpone retirement, measured in euros. Pension wealth includes only earnings‐related pensions. The values are in year 2000 euros and extreme values are excluded.

**FIGURE A4:**
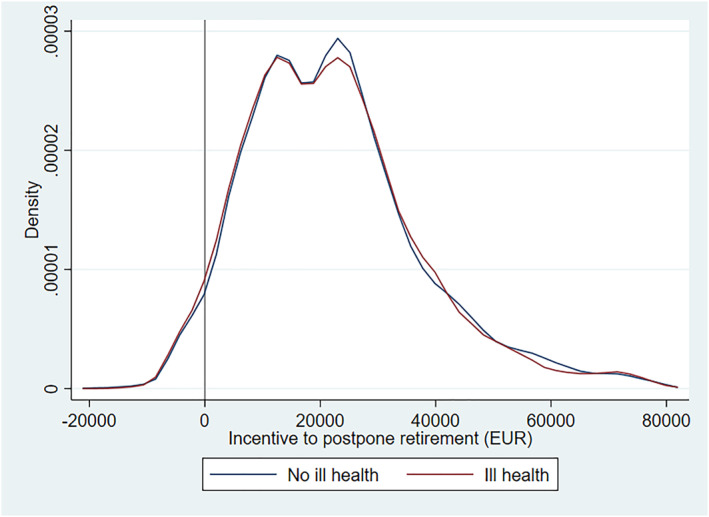
The distribution of retirement incentives (EUR) by ill health. The figure displays the distribution of the change in the amount of pension wealth when retirement is postponed by 1 year that is the financial incentive to postpone retirement, measured in euros. Pension wealth includes both earnings‐related pensions and national pensions. The values are in year 2000 euros and extreme values are excluded.

**FIGURE A5:**
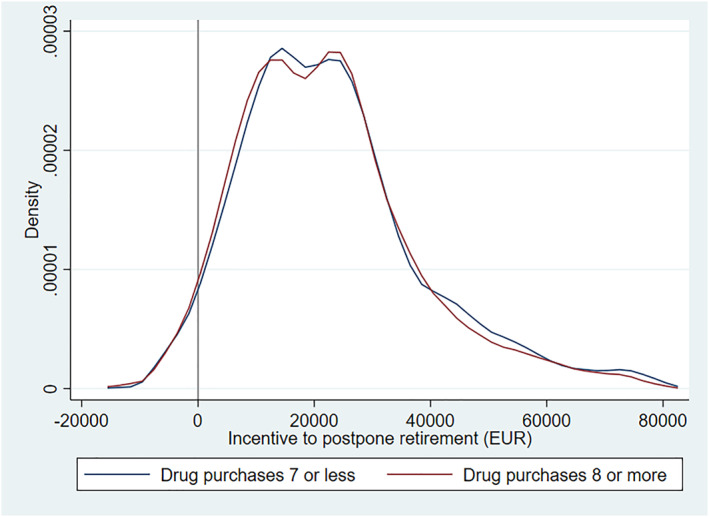
The distribution of retirement incentives (EUR) by medication purchases. The figure displays the distribution of the change in the amount of pension wealth when retirement is postponed by 1 year that is the financial incentive to postpone retirement, measured in euros. Pension wealth includes both earnings‐related pensions and national pensions. The values are in year 2000 euros and extreme values are excluded.

**FIGURE A6:**
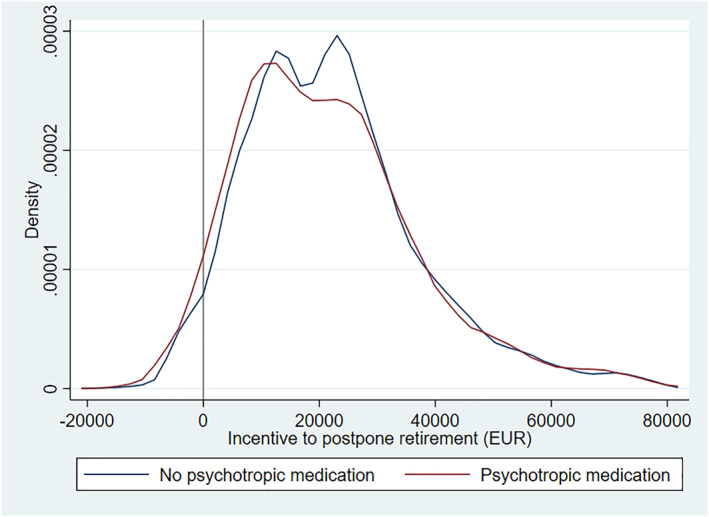
The distribution of retirement incentives (EUR) by psychotropic medication. The figure displays the distribution of the change in the amount of pension wealth when retirement is postponed by 1 year that is the financial incentive to postpone retirement, measured in euros. Pension wealth includes both earnings‐related pensions and national pensions. The values are in year 2000 euros and extreme values are excluded.

**FIGURE A7:**
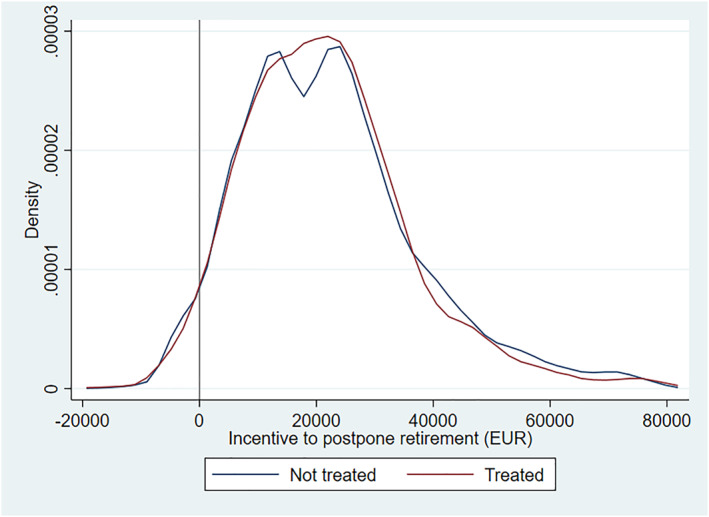
The distribution of retirement incentives (EUR) by any treatment. The figure displays the distribution of the change in the amount of pension wealth when retirement is postponed by 1 year that is the financial incentive to postpone retirement, measured in euros. Pension wealth includes both earnings‐related pensions and national pensions. The values are in year 2000 euros and extreme values are excluded.

**FIGURE A8:**
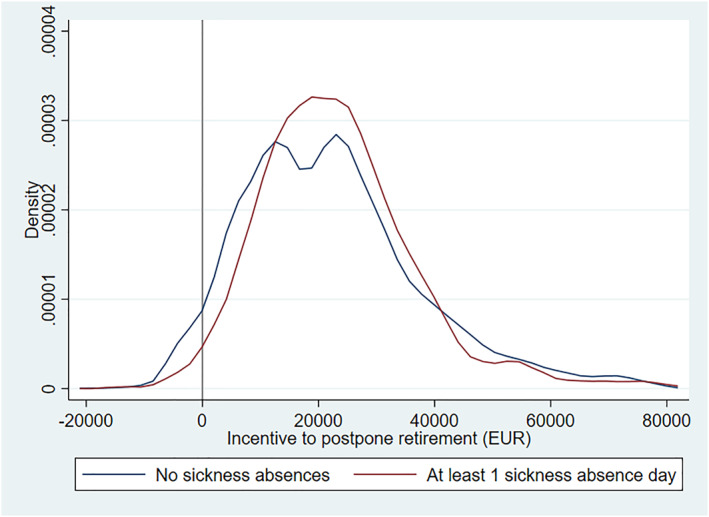
The distribution of retirement incentives (EUR) by any sickness absences. The figure displays the distribution of the change in the amount of pension wealth when retirement is postponed by 1 year that is the financial incentive to postpone retirement, measured in euros. Pension wealth includes both earnings‐related pensions and national pensions. The values are in year 2000 euros and extreme values are excluded.

**FIGURE A9:**
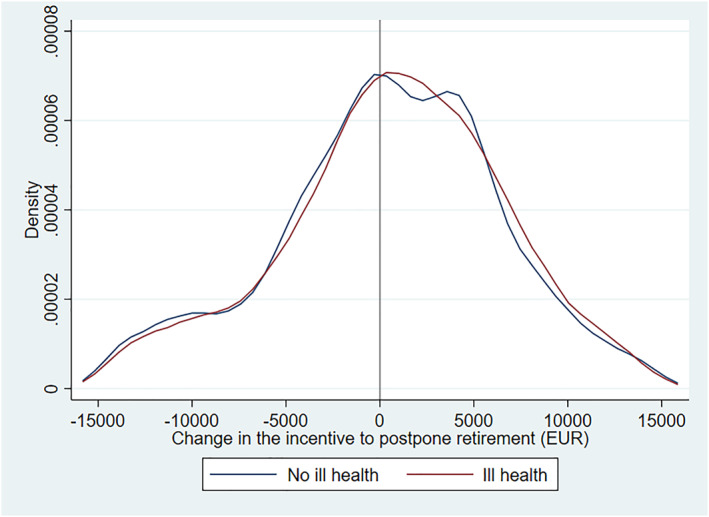
The distribution of the change in retirement incentives (EUR) in the 2005 reform, by ill health. The figure displays the distribution of the change implied by the 2005 reform in the financial incentive to postpone retirement, measured in euros. Pension wealth includes both earnings‐related pensions and national pensions. The values are in year 2000 euros and extreme values are excluded.

**FIGURE A10:**
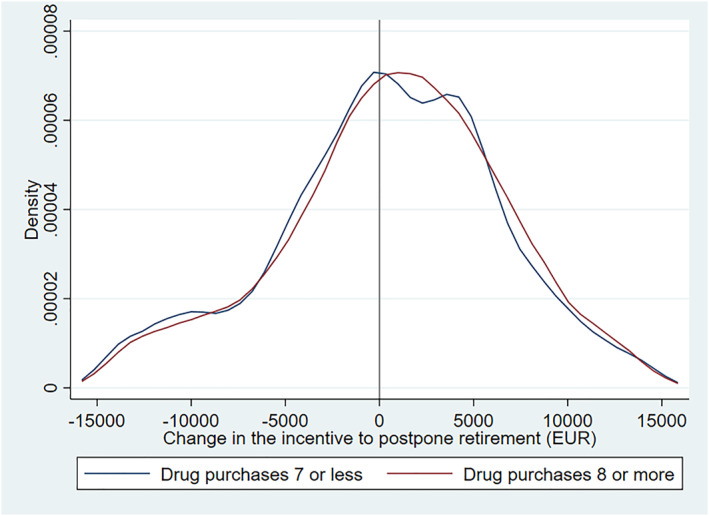
The distribution of the change in retirement incentives (EUR) in the 2005 reform, by medication purchases. The figure displays the distribution of the change implied by the 2005 reform in the financial incentive to postpone retirement, measured in euros. Pension wealth includes both earnings‐related pensions and national pensions. The values are in year 2000 euros and extreme values are excluded.

**FIGURE A11:**
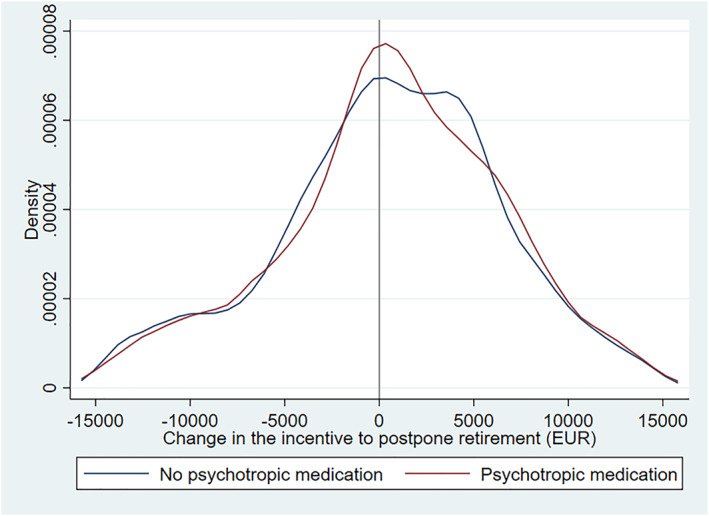
The distribution of the change in retirement incentives (EUR) in the 2005 reform, by psychotropic medication. The figure displays the distribution of the change implied by the 2005 reform in the financial incentive to postpone retirement, measured in euros. Pension wealth includes both earnings‐related pensions and national pensions. The values are in year 2000 euros and extreme values are excluded.

**FIGURE A12:**
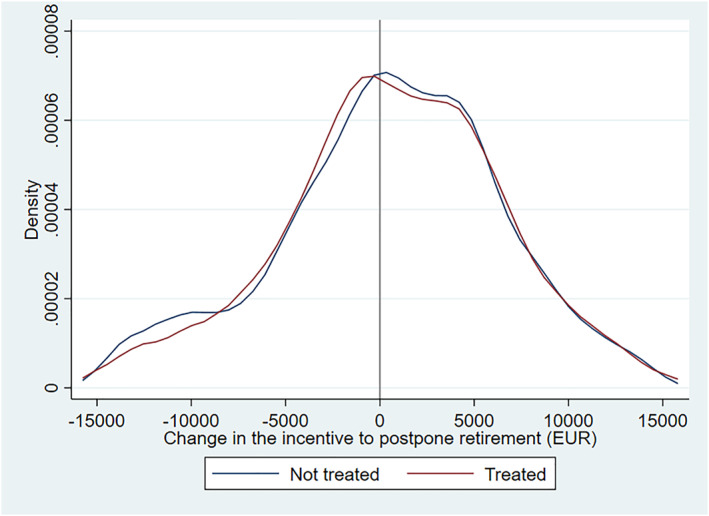
The distribution of the change in retirement incentives (EUR) in the 2005 reform, by any treatment. The figure displays the distribution of the change implied by the 2005 reform in the financial incentive to postpone retirement, measured in euros. Pension wealth includes both earnings‐related pensions and national pensions. The values are in year 2000 euros and extreme values are excluded.

**FIGURE A13:**
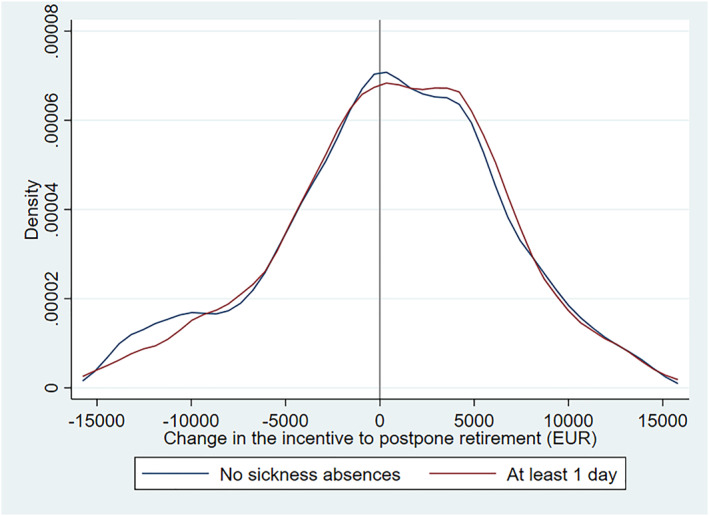
The distribution of the change in retirement incentives (EUR) in the 2005 reform, by sickness absences. The figure displays the distribution of the change implied by the 2005 reform in the financial incentive to postpone retirement, measured in euros. Pension wealth includes both earnings‐related pensions and national pensions. The values are in year 2000 euros and extreme values are excluded.

## Data Availability

Due to data protection regulations of the national register‐holders providing the data, we are not allowed to make the data available to third parties. Interested researchers have the possibility to obtain data access by contacting the following register‐holding public institutions: Statistics Finland (http://www.stat.fi/tup/mikroaineistot/index_en.html). Contact by email tutkijapalvelut@stat.fi. Findata (https://findata.fi/en/). Contact by email info(at)findata.fi. Findata handles applications for health data provided by the Finnish Institute for Health and Welfare, and the Social Insurance Institution. Finnish Centre for Pensions (https://www.etk.fi/en/).

## Endnotes

^1^
Gustman and Steinmeier (2018) also examine how health influences the role of economic incentives for the decision to retire and found that health does not affect responses to retirement incentives. However, their approach is very different to ours. They build a structural labour supply model with retirement and then simulate the impacts of changing incentives on people retiring, whereas we use a quasi‐experimental setting to obtain causal estimates of how incentives influence retirement decisions among people with different health status.

^2^
In a somewhat different context, Hall et al. (2022) analyzed individual differences in reactions to an active labour market programme targeted at young people, and found that young individuals who are in a difficult overall life situation (e.g. school dropouts and individuals suffering from mental health problems) do not react to activation. This implies that active labour market policies may not help individuals at risk of social exclusion. Since health problems are more relevant for the elderly, studying related issues in the context of pension reforms appears particularly relevant.

^3^
50% consumer price index and 50% earnings index.

^4^
80% consumer price index and 20% earnings index.

^5^
20% consumer price index and 80% earnings index.

^6^
The reform did not change the eligibility age for full national pension or the penalty for early claiming within that system.

^7^
Other measures for the incentive to stay in the labour force used in the literature are the option value measure (see Stock and Wise (1990)) and the peak value of pension wealth (see Coile and Gruber (2007)). The option value is formed by calculating the individual's utility at different retirement dates. These levels of utilities are compared to that with the highest utility (optimal retirement date) and this difference gives the measure for the incentive to retire at any given point in time. We choose not to use this measure, because it would require making additional assumptions about individuals' risk aversion and consumption and leisure preferences. The peak value is similar to the option value, without the need for making additional assumptions about individual preferences. The peak value approach compares the highest level of pension wealth with the current‐year level of pension wealth. One potential worry with the peak value measure is that it does not take into account how many years are needed to postpone retirement to reach the optimal level of pension wealth.

^8^
Before 2005, pensions were tied to the halfway index (for under 65‐year‐olds) and to the earnings‐related pension index (those 65 or older) and after that only to the earnings‐related pension index. National pensions are tied to the national pension index (before and after the reform).

^9^
For the first one we use 1.59%, for the second one we use 3.13% and for the third one 1.8%. We also convert pension wealth into year 2000 euros using the consumer price index.

^10^
Appendix: The Effect of the Reform on Incentives According to Health Statuses (Figures A2 and A3) contains some descriptive figures with earnings‐related pensions only.

^11^
In Appendix C (Figures A4, A5, A6, A7, A8, A9, A10, A11, A12, A13) we show how the incentives and the effect of the reform on incentives vary according to health status. The key takeaway from these charts is that the changes in incentives are very similar across individuals with different health status, and therefore it is unlikely that any difference in reactions to incentives between healthy and unhealthy individuals would stem from facing different incentives.

^12^
We identify hospitalisations and treatments due to diseases of the circulatory system (I00‐I99) and diseases of the musculoskeletal system and connective tissue (M00‐M99).

^13^
Medication with Anatomical Therapeutic Chemical (ATC) classification codes relating to psycholeptics (N05) and psychoanaleptics (N06, excluding anti‐dementia drugs N06D) were classified as psychotropic medication in the data.

^14^
Sickness absences that continue into the next year are allocated to both years according to the respective number of days.

^15^
These data do not cover information on occupational healthcare. However, this is unlikely to be a major limitation, given that the role of occupational healthcare in specialized care is non‐existent.

^16^
A full description of the control variables used in the estimations is presented in Appendix A.

^17^
We use also another instrument were the earnings are lagged by 2 years, formally $\ln\left(PW_{t+1}\left(y_{t-1},\text{…}\right)\right)-\ln\left(PW_{t}\left(y_{t-2},\text{…}\right)\right)$. Longer lags are more likely to be unaffected by possible mean reversion issues (Weber 2014): They are unrelated to the error term when there is first‐order autocorrelation.

^18^
Before the reform, reaching full retirement age was assigned value one when an individual turned 65. After the reform, it was assigned value one when an individual turned 63. In our analysis, year 2005 is excluded, so after the reform, only individuals turning 63 receive value one for the FRA variable. (In year 2005, individuals turning 63, 64, and 65 would have received value one for the FRA variable.)

^19^
On the other hand, the estimated coefficient for any treatment period, a treatment period due to musculoskeletal issues, a treatment period due to cardiovascular diseases, less than 8 medical purchases and having a length of sickness absences of 15–60 days are not statistically significant.

^20^
Both employed and unemployed persons are entitled to sickness allowance in Finland, but because the level of compensation is the same in both benefits, the unemployed may not be motivated to apply for sickness allowance.

## Appendix A Variables

The full list of control variables used in the estimations is presented here.

*Individual controls*: Gender, age at the end of the year, working history (≤ 35 years, 35–40 years, at least 40 years), level of education, field of education, region, reached full retirement age (yes or no), reached early retirement age (yes or no) and language. *Spouse controls*: Spouse (yes or no), age of spouse over early retirement age (yes or no) and age of spouse over full retirement age (yes or no). *Health controls*: prescriptive drug purchases (0–3, 4 to 7 and 8 or more), purchase of psychotropic medication (yes or no), any treatment period (yes or no), treatment period for circulatory or musculoskeletal diseases (yes or no), sickness absences (1–14 days, 15–60 days and over 60 days).

## Appendix B Further Robustness Checks

### Additional Description of the Changes in Incentives

Figure A1 shows the direct effect of the reform, in percentages, on pension wealth and incentives for those aged 60 to 66. The direct effect on pension wealth is calculated using the 2004 data and the information on total accrued pensions at the end of year 2004. As can be seen from the figure, people below the age of 65 saw an overnight increase in their pension wealth, since the eligibility age for old‐age pension decreased from 65 to 63 years of age. The effect was the highest for 63‐year‐old individuals, who saw an increase of around 9% in their pension wealth.^22^ The pensions of individuals who were at least 65 years old at the time of the reform were protected. Therefore, the change in pension wealth is zero in the figure for those age groups.

In addition to the overnight effects shown in Figure A1, the reform also had other effects on pension wealth. The larger accrual rate for those aged 53–60 years or at least 63 years, the change in the indexation of accrued pensions and the new feature that pensions accruing after the age of 63 did not affect national pensions all contributed to increase pension wealth. On the other hand, the smaller accrual rate for those aged 60–62 years, the change in delayed claiming, and the introduction of the life‐expectancy coefficient had a decreasing effect on pension wealth.

The log‐difference of pension wealth (incentives to postpone retirement) changed as well. The reform increased the incentives for those under 62.5 years, weakened incentives for those between 62.5 and 65 years, while the change in incentives was close to zero for those over 65 years of age.

### Robustness: Different Incentive Measures

In our baseline analysis, pension wealth was calculated using current earnings for those who were not retired or retiring in the current year. In column 5 of Table 5 we have tested how the results change if the pension wealth is calculated with 1 year lagged income for all individuals. Again the results are nearly identical with our main analysis indicating that the choice of income reference year does not drive the results. Similarly, in the subgroup analysis, the results closely resemble those of our main analysis (shown in Table A8).

In our main analysis we formed the measure for the incentives using changes in pension wealth, which is the discounted stream of future pensions until age 100. One possible issue with our choice is that the time horizon is too long. To test for the robustness of our results against this choice, we run the analysis using the other extreme, that is using annual pension instead of pension wealth, and using the accrual rate of the pension as the measure for the incentives to postpone retirement.

The results are shown in column 6 of Table 5 and they are very similar to the results of our baseline analysis. For the subgroup analysis, the results are also very similar (shown in Table A1).

### Sub‐Group Analysis Using Interaction Terms

In our primary analysis, we examined the variations in reactions to incentives across different health statuses by dividing the sample into distinct subgroups. In this section, we further evaluate the robustness of our findings by introducing interaction terms involving incentives, pension wealth, reaching full retirement age, and health variables. Formally, we estimate the following equation using OLS:

(A1)
$$\begin{array}{cc} R_{i,t}= & \theta_{1}\Delta \;\ln\left(PW_{i,t}\right)+\theta_{2}\Delta \;\ln\left(PW_{i,t}\right)\times {\text{Health}}_{i,t-1}+\theta_{3}\;\ln\left(PW_{i,t}\right)\\ & +\theta_{4}\;\ln\left(PW_{i,t}\right)\times {\text{Health}}_{i,t-1}+\theta_{5}FRA+\theta_{6}FRA\times {\text{Health}}_{i,t-1}\\ & +\theta_{7}{\text{Health}}_{i,t-1}+\gamma_{t}+\beta X_{i,t}+u_{i,t} \end{array}$$

where ${\text{Health}}_{i,t-1}$ is an indicator variable for having ill health (in the same domains as earlier) for individual i and all the other variables are defined in the same manner as in Equation (3). For this analysis, we employ the same set of health indicators as used in our primary subgroup analysis.

Table A2 presents the results of our subgroup analysis with interaction terms. The first three rows illustrate the impact of incentives, pension wealth, and reaching full retirement age on the retirement risk for individuals without the specific health status. Subsequently, the following three interactions describe how much the impact of these variables differs for individuals with the particular health status. Therefore, the total effect of these variables for individuals with the specific health status is the sum of these two effects. The last row demonstrates the impact of the specific health issue on the risk of retirement.

The findings in Table A2 support our earlier results. According to the interaction terms, the only statistically significant difference emerges among individuals with a sickness absence spell in the previous year. This group appears to be less responsive to incentives compared to those without such absence spells. The results indicate that a 1% point increase in incentives reduces the risk of retirement by approximately 1.4% points (calculated as −2.326 + 0.893) for individuals with sickness absence spells, while the estimated effect remains at −2.326 for those without such absence spells.

## Appendix D Additional Tables

**TABLE A1:** OLS estimates by population groups with pension and accrual rate of pension.

	Treatment, cardio (*t* − 1)		Treatment, muscular (*t* − 1)		Any treatment (*t* − 1)		Medication (*t* − 1)		Psychotropic medication (*t* − 1)		Sickness absences (*t* − 1)		Ill health (*t* − 1)	
	Yes	No	Yes	No	Yes	No	8≤	7 or less	Yes	No	1≤	No	Yes	No
Dependent variable is retirement decision														
Δln(pension)	−3.116**	−2.206***	−2.853***	−2.219***	−2.364***	−2.223***	−2.153***	−2.265***	−2.344***	−2.216***	−1.105**	−2.351***	−2.293***	−2.188***
	(1.001)	(0.127)	(0.852)	(0.128)	(0.366)	(0.134)	(0.208)	(0.159)	(0.398)	(0.133)	(0.418)	(0.132)	(0.195)	(0.166)
ln(pension)	−0.125	0.0514***	0.0523	0.0493***	0.0262	0.0524***	0.0535**	0.0435**	0.0624	0.0479***	0.0431	0.0501***	0.0523**	0.0441**
	(0.0808)	(0.0110)	(0.0942)	(0.0109)	(0.0344)	(0.0115)	(0.0184)	(0.0136)	(0.0380)	(0.0114)	(0.0351)	(0.0115)	(0.0176)	(0.0140)
FRA	0.253*	0.237***	0.0454	0.241***	0.185***	0.242***	0.254***	0.227***	0.219***	0.237***	0.156***	0.248***	0.253***	0.227***
	(0.101)	(0.0129)	(0.114)	(0.0128)	(0.0423)	(0.0134)	(0.0219)	(0.0157)	(0.0476)	(0.0133)	(0.0378)	(0.0135)	(0.0206)	(0.0162)
*p*‐value Δln(pension)	0.316		0.418		0.713		0.667		0.756		0.004		0.68	
*p*‐value ln(pension)	0.016		0.972		0.463		0.658		0.708		0.847		0.712	
*p*‐value FRA	0.861		0.06		0.191		0.303		0.706		0.02		0.307	
Observations	287	12,636	313	12,610	1447	11,476	4501	8422	1088	11,835	1543	11,380	4990	7933
R‐squared	0.438	0.319	0.412	0.320	0.320	0.322	0.312	0.327	0.312	0.324	0.343	0.320	0.313	0.327
FE	YES	YES	YES	YES	YES	YES	YES	YES	YES	YES	YES	YES	YES	YES
Individual controls	YES	YES	YES	YES	YES	YES	YES	YES	YES	YES	YES	YES	YES	YES
Spouse controls	YES	YES	YES	YES	YES	YES	YES	YES	YES	YES	YES	YES	YES	YES
Health controls *t* − 1	NO	NO	NO	NO	NO	NO	NO	NO	NO	NO	NO	NO	NO	NO

*Note:* The null hypothesis for the *p*‐values is that the difference between coefficients equals zero. Δln(pension) is the relative change in pension when retirement is postponed by 1 year. Sickness absences indicates days after the waiting period. Robust standard errors in parentheses ****p* < 0.001, ***p* < 0.01, **p* < 0.05.

**TABLE A2:** Sub‐group analysis (OLS) utilizing interaction terms.

	Treatment, cardio (*t* − 1)	Treatment, muscular (*t* − 1)	Any treatment (*t* − 1)	Medication (*t* − 1) (8 or more)	Psychotropic	Sickness	Ill health (*t* − 1)
					Medication (*t* − 1)	Absences (*t* − 1)	
Dependent variable is retirement decision							
Δln(PW)	−2.213***	−2.231***	−2.236***	−2.286***	−2.223***	−2.326***	−2.213***
	(0.127)	(0.127)	(0.133)	(0.153)	(0.132)	(0.131)	(0.158)
ln(PW)	0.0493***	0.0463***	0.0482***	0.0401***	0.0461***	0.0461***	0.0396**
	(0.0109)	(0.0109)	(0.0111)	(0.0121)	(0.0110)	(0.0111)	(0.0123)
FRA	0.237***	0.237***	0.237***	0.227***	0.238***	0.239***	0.228***
	(0.0128)	(0.0128)	(0.0131)	(0.0142)	(0.0130)	(0.0131)	(0.0145)
Δln(PW)× ${Health}_{t-1}$	−0.776	0.110	0.0883	0.177	−0.0213	0.893*	−0.0133
	(0.784)	(0.748)	(0.344)	(0.233)	(0.371)	(0.379)	(0.228)
ln(PW)× ${Health}_{t-1}$	−0.0575	0.0986	0.000111	0.0212	0.0269	0.0278	0.0204
	(0.0493)	(0.0557)	(0.0240)	(0.0154)	(0.0276)	(0.0263)	(0.0151)
FRA × ${Health}_{t-1}$	−0.0184	−0.0144	0.000612	0.0302	−0.00885	−0.0194	0.0241
	(0.0568)	(0.0580)	(0.0276)	(0.0182)	(0.0313)	(0.0275)	(0.0178)
${Health}_{t-1}$	0.797	−1.205	0.000491	−0.264	−0.293	−0.371	−0.238
	(0.613)	(0.692)	(0.297)	(0.190)	(0.341)	(0.324)	(0.186)
Observations	12,923	12,923	12,923	12,923	12,923	12,923	12,923
R‐squared	0.319	0.320	0.319	0.320	0.320	0.320	0.320
FE	YES	YES	YES	YES	YES	YES	YES
Individual controls	YES	YES	YES	YES	YES	YES	YES
Spouse controls	YES	YES	YES	YES	YES	YES	YES
Health controls *t* − 1	NO	NO	NO	NO	NO	NO	NO

*Note:* ${\text{Health}}_{t-1}$ is an indicator variable representing various health statuses, with its interpretation varying across each model. Δln(PW) is the financial incentives to postpone retirement (i.e. the relative change in pension wealth, PW, when retirement is postponed by 1 year). FRA is an indicator for reaching full retirement age. The interaction terms, Δln(PW)× ${\text{Health}}_{t-1}$, ln(PW)× ${\text{Health}}_{t-1}$, and FRA × ${\text{Health}}_{t-1}$, capture the interplay between incentives, pension wealth, reaching full retirement age, and health variables. Interaction terms depict the situation where health indicator gets the value 1. In interaction term, FRA × ${\text{Health}}_{t-1}$, FRA also gets the value 1. Sickness absences indicates days after the waiting period. Robust standard errors in parentheses ****p* < 0.001, ***p* < 0.01, **p* < 0.05.

**TABLE A3:** Descriptive statistics by sickness absences.

	Sickness absences (*t* − 1)	
	No	1 ≤
Share	0.881	0.119
Retirement rate	0.286	0.262
Reaching full retirement age	0.265	0.255
Female	0.459	0.399
Age (at the end of the year)	63.01	63.05
Spouse (share)	0.715	0.690
Working history (years)	36.25	37.19
Unemployed, share (*t* − 1)	0.0874	0.0402
Tertiary degree, share	0.132	0.0726
Earnings	24,705	23,702
Accrued pension (euros)	15,791	14,653
Pension wealth (euros)	328,858	304,527
Pension wealth (logs)	12.57	12.54
Δln(PW)	0.0667	0.0710
Δ(PW)	23,508	22,748
Psychotropic medication (*t* − 1)	0.0752	0.150
Medication purchases (*t* − 1)	6.127	10.06
Any treatment (*t* − 1)	0.0570	0.517
Treatment, cardio (*t* − 1)	0.011	0.106
Treatment, muscular (*t* − 1)	0.00691	0.151

*Note:* Monetary values are in year 2000 euros. Sickness absences in days refers to the length of sickness absence spells after the waiting period. The share of unemployed in the previous year is identified by using information about the main type of activity of an individual within a year.

**TABLE A4:** Descriptive statistics on retirement by gender and education.

	Men	Women	Low	Middle	High
Risk of DI retirement	2.3%	2.2%	2.8%	2.1%	0.9%
Risk of old‐age retirement	23.8%	19.0%	24.2%	23.9%	24.6%
Share of DI retirement	57.0%	43.0%	57.0%	38.0%	5.0%
Share of old‐age retirement	55.6%	44.4%	45.5%	41.2%	13.3%
Share of population	56.3%	43.7%	45.4%	41.6%	13.1%

*Note:* Risk of DI retirement and risk of old‐age retirement indicate retirement risks based on different characteristic. The share of DI retirement, the share of old‐age retirement, and the share of the population illustrate the distribution of various characteristics among retirees and the population. Before the reform individuals aged 62 to 65 are included. After the reform individuals aged 62 to 63 are included. Low, middle and high refer to educational level. All working sectors are included.

**TABLE A5:** Descriptive statistics on retirement by year.

Year	2001	2002	2003	2004	2005	2006	2007	2008	2009
Old‐age retirement	22.1%	20.6%	15.1%	14.8%	31.0%	28.2%	25.3%	27.9%	31.1%
Disability retirement	3.3%	3.9%	3.9%	2.6%	2.0%	1.7%	1.4%	1.4%	1.3%

*Note:* Before the reform individuals aged 62 to 65 are included. After the reform individuals aged 62 to 63 are included. All working sectors are included.

**TABLE A6:** OLS estimates by population groups, excluding individuals entitled to national pensions.

	Treatment, cardio (*t* − 1)		Treatment, muscular (*t* − 1)		Any treatment (*t* − 1)		Medication (*t* − 1)		Psychotropic medication (*t* − 1)		Sickness absences (*t* − 1)		Ill health (*t* − 1)	
	Yes	No	Yes	No	Yes	No	8≤	7 or less	Yes	No	1≤	No	Yes	No
Dependent variable is retirement decision														
Δln(PW)	−4.928***	−2.637***	−2.879*	−2.688***	−2.543***	−2.688***	−2.726***	−2.636***	−2.909***	−2.661***	−1.187	−2.803***	−2.858***	−2.531***
	(1.293)	(0.177)	(1.431)	(0.177)	(0.561)	(0.184)	(0.287)	(0.223)	(0.563)	(0.186)	(0.632)	(0.183)	(0.269)	(0.232)
ln(PW)	−0.261*	0.0669***	0.130	0.0612***	0.0336	0.0647***	0.0502	0.0656***	0.0424	0.0626***	0.0583	0.0609***	0.0438	0.0706***
	(0.117)	(0.0160)	(0.134)	(0.0159)	(0.0480)	(0.0167)	(0.0269)	(0.0196)	(0.0583)	(0.0165)	(0.0507)	(0.0167)	(0.0257)	(0.0201)
FRA	0.241	0.254***	0.172	0.252***	0.212***	0.255***	0.225***	0.259***	0.119	0.260***	0.185***	0.261***	0.227***	0.263***
	(0.139)	(0.0164)	(0.153)	(0.0164)	(0.0570)	(0.0170)	(0.0311)	(0.0197)	(0.0720)	(0.0168)	(0.0502)	(0.0173)	(0.0292)	(0.0201)
*p*‐value Δln(PW)	0.039		0.877		0.801		0.802		0.663		0.012		0.353	
*p*‐value ln(PW)	0.001		0.553		0.529		0.642		0.728		0.959		0.407	
*p*‐value FRA	0.912		0.545		0.459		0.346		0.046		0.137		0.305	
Observations	194	8182	193	8183	918	7458	2859	5517	642	7734	976	7400	3158	5218
R‐squared	0.484	0.325	0.511	0.325	0.323	0.329	0.317	0.333	0.348	0.328	0.359	0.325	0.318	0.335
FE	YES	YES	YES	YES	YES	YES	YES	YES	YES	YES	YES	YES	YES	YES
Individual controls	YES	YES	YES	YES	YES	YES	YES	YES	YES	YES	YES	YES	YES	YES
Spouse controls	YES	YES	YES	YES	YES	YES	YES	YES	YES	YES	YES	YES	YES	YES
Health controls *t* − 1	NO	NO	NO	NO	NO	NO	NO	NO	NO	NO	NO	NO	NO	NO

*Note:* The null hypothesis for the *p*‐values is that the difference between coefficients equals zero. Δln(PW) is the financial incentives to postpone retirement (i.e. the relative change in pension wealth, PW, when retirement is postponed by 1 year). Sickness absences indicates days after the waiting period. Those entitled to national pensions are excluded from the analysis. Robust standard errors in parentheses ****p* < 0.001, ***p* < 0.01, **p* < 0.05.

**TABLE A7:** OLS estimates by population groups, excluding unemployed.

	Treatment, cardio (*t* − 1)		Treatment, muscular (*t* − 1)		Any treatment (*t* − 1)		Medication (*t* − 1)		Psychotropic medication (*t* − 1)		Sickness absences (*t* − 1)		Ill health (*t* − 1)	
	Yes	No	Yes	No	Yes	No	8≤	7 or less	Yes	No	1≤	No	Yes	No
Dependent variable is retirement decision														
Δln(PW)	−2.448*	−1.288***	−3.037**	−1.285***	−1.876***	−1.250***	−1.195***	−1.383***	−1.477**	−1.297***	−0.889*	−1.375***	−1.394***	−1.271***
	(1.074)	(0.138)	(0.981)	(0.138)	(0.401)	(0.146)	(0.225)	(0.173)	(0.457)	(0.144)	(0.447)	(0.144)	(0.212)	(0.180)
ln(PW)	−0.158	0.0446***	0.0441	0.0423***	0.0126	0.0464***	0.0489*	0.0362**	0.0382	0.0428***	0.0424	0.0427***	0.0453*	0.0378**
	(0.0836)	(0.0114)	(0.0982)	(0.0113)	(0.0362)	(0.0118)	(0.0190)	(0.0140)	(0.0414)	(0.0117)	(0.0360)	(0.0118)	(0.0182)	(0.0144)
FRA	0.273*	0.240***	0.0612	0.244***	0.179***	0.246***	0.256***	0.230***	0.221***	0.240***	0.151***	0.252***	0.254***	0.231***
	(0.112)	(0.0135)	(0.116)	(0.0134)	(0.0454)	(0.0140)	(0.0228)	(0.0166)	(0.0512)	(0.0139)	(0.0391)	(0.0142)	(0.0216)	(0.0170)
*p*‐value Δln(PW)	0.227		0.051		0.135		0.505		0.699		0.292		0.656	
*p*‐value ln(PW)	0.007		0.983		0.366		0.589		0.913		0.994		0.745	
*p*‐value FRA	0.736		0.085		0.145		0.357		0.718		0.013		0.397	
Observations	258	11,604	299	11,563	1331	10,531	4124	7738	967	10,895	1481	10,381	4550	7312
R‐squared	0.457	0.314	0.407	0.315	0.302	0.318	0.311	0.320	0.304	0.319	0.340	0.314	0.310	0.321
FE	YES	YES	YES	YES	YES	YES	YES	YES	YES	YES	YES	YES	YES	YES
Individual controls	YES	YES	YES	YES	YES	YES	YES	YES	YES	YES	YES	YES	YES	YES
Spouse controls	YES	YES	YES	YES	YES	YES	YES	YES	YES	YES	YES	YES	YES	YES
Health controls *t* − 1	NO	NO	NO	NO	NO	NO	NO	NO	NO	NO	NO	NO	NO	NO

*Note:* The null hypothesis for the *p*‐values is that the difference between coefficients equals zero. Δln(PW) is the financial incentives to postpone retirement (i.e. the relative change in pension wealth, PW, when retirement is postponed by 1 year). Sickness absences indicates days after the waiting period. Individuals who were mainly unemployed in the previous year are excluded from the analysis. Robust standard errors in parentheses ****p* < 0.001, ***p* < 0.01, **p* < 0.05.

**TABLE A8:** OLS estimates by population groups, 1 year lagged income.

	Treatment, cardio (*t* − 1)		Treatment, muscular (*t* − 1)		Any treatment (*t* − 1)		Medication (*t* − 1)		Psychotropic medication (*t* − 1)		Sickness absences (*t* − 1)		Ill health (*t* − 1)	
	Yes	No	Yes	No	Yes	No	8≤	7 or less	Yes	No	1≤	No	Yes	No
Dependent variable is retirement decision														
Δln(PW)	−3.548***	−2.267***	−2.848***	−2.283***	−2.335***	−2.296***	−2.249***	−2.308***	−2.358***	−2.285***	−1.195**	−2.411***	−2.393***	−2.228***
	(1.006)	(0.126)	(0.847)	(0.127)	(0.357)	(0.134)	(0.207)	(0.158)	(0.400)	(0.132)	(0.422)	(0.131)	(0.193)	(0.165)
ln(PW)	−0.130	0.0526***	0.0556	0.0505***	0.0254	0.0538***	0.0551**	0.0445***	0.0631	0.0491***	0.0440	0.0513***	0.0540**	0.0449**
	(0.0798)	(0.0109)	(0.0929)	(0.0108)	(0.0339)	(0.0114)	(0.0182)	(0.0134)	(0.0376)	(0.0113)	(0.0348)	(0.0113)	(0.0174)	(0.0138)
FRA	0.247*	0.237***	0.0482	0.240***	0.183***	0.242***	0.253***	0.227***	0.217***	0.237***	0.155***	0.247***	0.252***	0.227***
	(0.100)	(0.0128)	(0.113)	(0.0128)	(0.0422)	(0.0134)	(0.0219)	(0.0157)	(0.0476)	(0.0133)	(0.0378)	(0.0135)	(0.0206)	(0.0162)
*p*‐value Δln(PW)	0.16		0.468		0.917		0.819		0.86		0.005		0.513	
*p*‐value ln(PW)	0.012		0.952		0.418		0.637		0.715		0.839		0.681	
*p*‐value FRA	0.912		0.064		0.177		0.322		0.689		0.02		0.327	
Observations	287	12,636	313	12,610	1447	11,476	4501	8422	1088	11,835	1543	11,380	4990	7933
R‐squared	0.445	0.321	0.413	0.322	0.320	0.323	0.314	0.328	0.313	0.326	0.344	0.322	0.315	0.328
FE	YES	YES	YES	YES	YES	YES	YES	YES	YES	YES	YES	YES	YES	YES
Individual controls	YES	YES	YES	YES	YES	YES	YES	YES	YES	YES	YES	YES	YES	YES
Spouse controls	YES	YES	YES	YES	YES	YES	YES	YES	YES	YES	YES	YES	YES	YES
Health controls *t* − 1	NO	NO	NO	NO	NO	NO	NO	NO	NO	NO	NO	NO	NO	NO

*Note:* The null hypothesis for the *p*‐values is that the difference between the coefficients equals zero. Δln(PW) is the relative change in pension wealth when retirement is postponed by 1 year. Pension wealth is calculated using 1 year lagged incomes. Sickness absences indicates days after the waiting period. Robust standard errors in parentheses ****p* < 0.001, ***p* < 0.01, **p* < 0.05.

**TABLE A9:** OLS estimates by population groups including disability retirements.

	Treatment, cardio (*t* − 1)		Treatment, muscular (*t* − 1)		Any treatment (*t* − 1)		Medication (*t* − 1)		Psychotropic medication (*t* − 1)		Sickness absences (*t* − 1)		Ill health (*t* − 1)	
	Yes	No	Yes	No	Yes	No	8≤	7 or less	Yes	No	1≤	No	Yes	No
Dependent variable is retirement decision														
Δln(PW)	−3.103**	−2.125***	−3.477***	−2.129***	−2.468***	−2.118***	−2.043***	−2.214***	−1.997***	−2.165***	−1.827***	−2.273***	−2.200***	−2.103***
	(1.011)	(0.130)	(0.856)	(0.130)	(0.379)	(0.137)	(0.213)	(0.161)	(0.418)	(0.135)	(0.454)	(0.134)	(0.200)	(0.168)
ln(PW)	−0.165	0.0472***	0.0397	0.0451***	−0.00278	0.0498***	0.0419*	0.0436**	0.0506	0.0441***	−0.00404	0.0506***	0.0404*	0.0445**
	(0.0844)	(0.0112)	(0.0976)	(0.0111)	(0.0353)	(0.0116)	(0.0187)	(0.0138)	(0.0391)	(0.0116)	(0.0380)	(0.0115)	(0.0180)	(0.0141)
FRA	0.200*	0.240***	0.0142	0.242***	0.166***	0.246***	0.243***	0.232***	0.212***	0.238***	0.140***	0.254***	0.245***	0.231***
	(0.0982)	(0.0131)	(0.112)	(0.0130)	(0.0433)	(0.0136)	(0.0226)	(0.0159)	(0.0486)	(0.0135)	(0.0389)	(0.0137)	(0.0213)	(0.0164)
*p*‐value Δln(PW)	0.292		0.09		0.378		0.52		0.696		0.339		0.709	
*p*‐value ln(PW)	0.006		0.952		0.15		0.941		0.871		0.163		0.855	
*p*‐value FRA	0.658		0.029		0.074		0.697		0.593		0.005		0.597	
Observations	325	12,919	348	12,896	1566	11,678	4691	8553	1166	12,078	1791	11,453	5245	7999
R‐squared	0.339	0.275	0.304	0.276	0.225	0.285	0.245	0.294	0.215	0.284	0.166	0.305	0.236	0.306
FE	YES	YES	YES	YES	YES	YES	YES	YES	YES	YES	YES	YES	YES	YES
Individual controls	YES	YES	YES	YES	YES	YES	YES	YES	YES	YES	YES	YES	YES	YES
Spouse controls	YES	YES	YES	YES	YES	YES	YES	YES	YES	YES	YES	YES	YES	YES
Health controls *t* − 1	NO	NO	NO	NO	NO	NO	NO	NO	NO	NO	NO	NO	NO	NO

*Note:* The null hypothesis for the *p*‐values is that the difference between coefficients equals zero.Δln(PW) is the financial incentives to postpone retirement (i.e., the relative change in pension wealth, PW, when retirement is postponed by 1 year). Sickness absences indicates days after the waiting period. Pension wealth and the incentives are calculated according to Equation (4). Robust standard errors in parentheses ****p* < 0.001, ***p* < 0.01, **p* < 0.05.

**TABLE A10:** 2SLS estimates by population groups (main sample), 1 year lagged income.

	Treatment, cardio (*t* − 1)		Treatment, muscular (*t* − 1)		Any treatment (*t* − 1)		Medication (*t* − 1)		Psychotropic medication (*t* − 1)		Sickness absences (*t* − 1)		Ill health (*t* − 1)	
	Yes	No	Yes	No	Yes	No	8≤	7 or less	Yes	No	1≤	No	Yes	No
Dependent variable is retirement decision														
Δln(PW)	−3.543***	−2.343***	−2.968***	−2.357***	−2.457***	−2.365***	−2.314***	−2.388***	−2.395***	−2.364***	−1.231**	−2.491***	−2.460***	−2.307***
	(0.910)	(0.130)	(0.801)	(0.130)	(0.367)	(0.137)	(0.210)	(0.163)	(0.394)	(0.136)	(0.428)	(0.135)	(0.197)	(0.170)
ln(PW)	−0.128	0.0513***	0.0514	0.0492***	0.0258	0.0523***	0.0531**	0.0435**	0.0615	0.0478***	0.0429	0.0499***	0.0520**	0.0440**
	(0.0723)	(0.0110)	(0.0849)	(0.0109)	(0.0335)	(0.0114)	(0.0182)	(0.0135)	(0.0368)	(0.0113)	(0.0343)	(0.0114)	(0.0174)	(0.0138)
FRA	0.246**	0.236***	0.0438	0.240***	0.185***	0.241***	0.253***	0.226***	0.218***	0.236***	0.155***	0.247***	0.252***	0.226***
	(0.0907)	(0.0128)	(0.103)	(0.0128)	(0.0415)	(0.0133)	(0.0217)	(0.0157)	(0.0464)	(0.0132)	(0.0371)	(0.0135)	(0.0205)	(0.0162)
*p*‐value Δln(PW)	0.192		0.451		0.815		0.783		0.939		0.005		0.557	
*p*‐value ln(PW)	0.015		0.979		0.455		0.672		0.721		0.845		0.72	
*p*‐value FRA	0.912		0.06		0.195		0.309		0.714		0.021		0.316	
Observations	287	12,636	313	12,610	1447	11,476	4501	8422	1088	11,835	1543	11,380	4990	7933
R‐squared	0.438	0.319	0.412	0.320	0.320	0.322	0.311	0.327	0.312	0.324	0.343	0.320	0.313	0.327
Kleibergen‐Paap	10,440	57,495	3490	57,772	1781	90,077	15,547	52,954	19,760	50,234	8639	52,018	18,590	47,410
*F* statistics														
First stage: Dependent variable is relative change in pension wealth calculated using 1 year lagged income $\left(\ln\left(PW_{t+1}\left(y_{t},\text{…}\right)\right)-\ln\left(PW_{t}\left(y_{t-1},\text{…}\right)\right)\right)$														
First stage	0.98	0.963	0.972	0.964	0.952	0.965	0.965	0.962	0.98	0.962	0.968	0.963	0.967	0.961
r‐squared														
FE	YES	YES	YES	YES	YES	YES	YES	YES	YES	YES	YES	YES	YES	YES
Individual controls	YES	YES	YES	YES	YES	YES	YES	YES	YES	YES	YES	YES	YES	YES
Spouse controls	YES	YES	YES	YES	YES	YES	YES	YES	YES	YES	YES	YES	YES	YES
Health controls *t* − 1	NO	NO	NO	NO	NO	NO	NO	NO	NO	NO	NO	NO	NO	NO

*Note:* The null hypothesis for the *p*‐values is that the difference between coefficients equals zero. Δln(PW) is the financial incentives to postpone retirement (i.e. the relative change in pension wealth, PW, when retirement is postponed by 1 year). Sickness absences indicates days after the waiting period. Robust standard errors in parentheses ****p* < 0.001, ***p* < 0.01, **p* < 0.05.

**TABLE A11:** 2SLS estimates by population groups, 2 year lagged income.

	Treatment, cardio (*t* − 1)		Treatment, muscular (*t* − 1)		Any treatment (*t* − 1)		Medication (*t* − 1)		Psychotropic medication (*t* − 1)		Sickness absences (*t* − 1)		Ill health (*t* − 1)	
	Yes	No	Yes	No	Yes	No	8≤	7 or less	Yes	No	1≤	No	Yes	No
Dependent variable is retirement decision														
Δln(PW)	−3.509***	−2.822***	−2.890**	−2.838***	−3.089***	−2.825***	−2.785***	−2.861***	−2.821***	−2.841***	−0.954	−3.029***	−2.921***	−2.794***
	(1.061)	(0.148)	(0.956)	(0.148)	(0.443)	(0.155)	(0.237)	(0.187)	(0.448)	(0.155)	(0.523)	(0.153)	(0.223)	(0.194)
ln(PW)	−0.139	0.0482***	0.0715	0.0456***	0.0354	0.0480***	0.0486*	0.0414**	0.0403	0.0459***	0.0494	0.0460***	0.0471**	0.0429**
	(0.0793)	(0.0116)	(0.0908)	(0.0115)	(0.0350)	(0.0121)	(0.0190)	(0.0143)	(0.0383)	(0.0120)	(0.0360)	(0.0120)	(0.0181)	(0.0148)
FRA	0.265**	0.238***	−0.0437	0.243***	0.198***	0.242***	0.256***	0.227***	0.264***	0.235***	0.149***	0.250***	0.257***	0.226***
	(0.0963)	(0.0139)	(0.128)	(0.0138)	(0.0446)	(0.0145)	(0.0238)	(0.0170)	(0.0490)	(0.0144)	(0.0412)	(0.0146)	(0.0225)	(0.0175)
*p*‐value Δln(PW)	0.522		0.957		0.574		0.801		0.966		< 0.001		0.669	
*p*‐value ln(PW)	0.02		0.777		0.733		0.76		0.888		0.927		0.856	
*p*‐value FRA	0.783		0.025		0.346		0.312		0.571		0.021		0.286	
Observations	264	11,448	282	11,430	1336	10,376	4157	7555	997	10,715	1401	10,311	4599	7113
R‐squared	0.446	0.304	0.410	0.305	0.310	0.306	0.300	0.310	0.320	0.307	0.330	0.305	0.302	0.310
Kleibergen‐Paap	234	12,117	528	12,058	801	12,807	6496	6707	594	12,884	859	11,364	4549	8047
*F* statistics														
First stage: Dependent variable is relative change in pension wealth calculated using 2 year lagged income $\left(\ln\left(PW_{t+1}\left(y_{t-1},\text{…}\right)\right)-\ln\left(PW_{t}\left(y_{t-2},\text{…}\right)\right)\right)$														
First stage	0.808	0.829	0.835	0.829	0.754	0.839	0.836	0.824	0.844	0.826	0.79	0.832	0.826	0.83
r‐squared														
FE	YES	YES	YES	YES	YES	YES	YES	YES	YES	YES	YES	YES	YES	YES
Individual controls	YES	YES	YES	YES	YES	YES	YES	YES	YES	YES	YES	YES	YES	YES
Spouse controls	YES	YES	YES	YES	YES	YES	YES	YES	YES	YES	YES	YES	YES	YES
Health controls *t* − 1	NO	NO	NO	NO	NO	NO	NO	NO	NO	NO	NO	NO	NO	NO

*Note:* The null hypothesis for the *p*‐values is that the difference between coefficients equals zero. Δln(PW) is the financial incentives to postpone retirement (i.e. the relative change in pension wealth, PW, when retirement is postponed by 1 year). Sickness absences indicates days after the waiting period. Robust standard errors in parentheses ****p* < 0.001, ***p* < 0.01, **p* < 0.05.
